# Binding site elucidation and structure guided design of macrocyclic IL-17A antagonists

**DOI:** 10.1038/srep30859

**Published:** 2016-08-16

**Authors:** Shenping Liu, Leslie A. Dakin, Li Xing, Jane M. Withka, Parag V. Sahasrabudhe, Wei Li, Mary Ellen Banker, Paul Balbo, Suman Shanker, Boris A. Chrunyk, Zuojun Guo, Jinshan M. Chen, Jennifer A. Young, Guoyun Bai, Jeremy T. Starr, Stephen W. Wright, Joerg Bussenius, Sheng Tan, Ariamala Gopalsamy, Bruce A. Lefker, Fabien Vincent, Lyn H. Jones, Hua Xu, Lise R. Hoth, Kieran F. Geoghegan, Xiayang Qiu, Mark E. Bunnage, Atli Thorarensen

**Affiliations:** 1 Worldwide Medicinal Chemistry, Pfizer Worldwide R&D, Eastern Point Road, Groton, CT 06340, USA; 2 Worldwide Medicinal Chemistry, Pfizer Worldwide R&D, 610 Main Street, Cambridge, MA 02139, USA; 3 Inflammation and Immunoscience Research Unit, Pfizer Worldwide R&D, 610 Main Street, Cambridge, MA 02139, USA; 4 Primary Pharmacology Group, Pfizer Worldwide R&D, Eastern Point Road, Groton, CT 06340, USA; 5 WuXi AppTec, 288 Fute Zhong Road, Waigaoqiao Free Trade Zone, Shanghai 200131, China

## Abstract

Interleukin-17A (IL-17A) is a principal driver of multiple inflammatory and immune disorders. Antibodies that neutralize IL-17A or its receptor (IL-17RA) deliver efficacy in autoimmune diseases, but no small-molecule IL-17A antagonists have yet progressed into clinical trials. Investigation of a series of linear peptide ligands to IL-17A and characterization of their binding site has enabled the design of novel macrocyclic ligands that are themselves potent IL-17A antagonists.

Interleukin-17 (IL-17) cytokines are homo or heterodimeric proteins formed by combinations of six distinct polypeptides designated IL17A-F^1^. They are essential to a fully functional immune system^2^,^3^, but dysregulated expression of IL-17A is implicated in autoimmune disorders such as psoriasis, psoriatic arthritis, rheumatoid arthritis and multiple sclerosis^4^,^5^,^6^. The IL-17A covalent homodimer’s significance in psoriasis is evidenced by the recent success of anti-IL-17A biologics as therapeutics. Secukinumab (Costentyx^TM^), a monoclonal antibody targeting IL-17A, was recently approved for the treatment of moderate to severe plaque psoriasis^7^,^8^ and is being investigated in other IL-17A-driven immunological diseases^9^. Additionally, two other biologics, ixekizumab (anti-IL17A)^10^,^11^ and brodalumab (an antibody to the IL-17 receptor, IL-17RA)^12^,^13^, have shown efficacy in psoriasis in late stage clinical trials.

IL-17A signaling occurs through its membrane-bound receptors, IL-17RA and IL-17RC, and elicits multiple inflammatory and immune responses^14^,^15^,^16^. The cytokine binds to IL-17RA with low single-digit nanomolar affinity^14^,^15^,^17^,^18^. and the structure of their complex is known^17^. The emerging biologics block this interaction by binding to one or other of the partners, but our goal was to determine whether it could be blocked or modulated with a small molecule as this could afford orally active agents.

Small-molecule inhibition of a protein-protein interaction (PPI) is invariably challenging^19^. Even the discovery of early lead matter tends to be difficult because corporate compound collections are largely designed to target the active centers of enzymes, and are deficient in compounds suitable to the longer and shallower binding sites on which PPIs tend to depend. As the industry expands the “druggable genome”, continued efforts at small molecule inhibition of PPIs will be required^20^.

## Results

### Lead small molecule IL-17A antagonists

**Figure 1:**
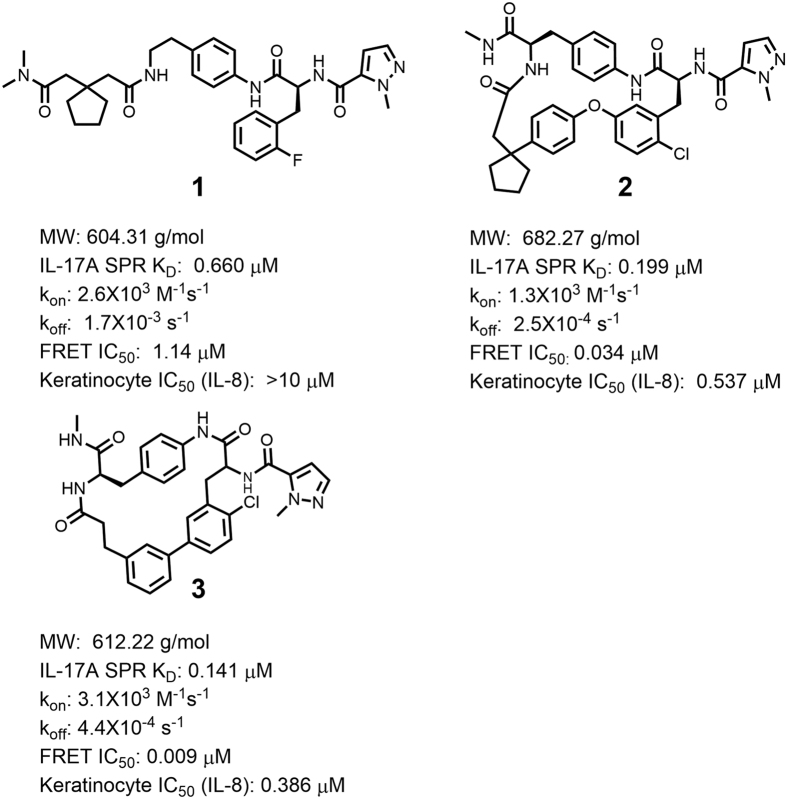
Chemical structures of example IL-17A inhibitors used in this study. Compound **1**: example of a lead IL-17A antagonist with a linear peptide motif. Compounds **2** and **3:** macrocyclic IL-17A antagonists designed on basis of the structure of compound **1** complexed with IL-17A.

Our effort to discover small-molecule antagonists of IL-17A was initiated from disclosed inhibitors^21^,^22^ exemplified by compound **1** (Fig. 1), a polyamide with clear structure-activity relationships (SAR) representative of the series. For example, the amide bonds, correct chiral center and cyclopentyl group were all required for activity. Surface plasmon resonance (SPR) measurements showed that compound **1** bound directly to IL-17A with a K_D_ of 0.66 μM. It also blocked the IL-17A/IL-17RA interaction in a fluorescence resonance energy transfer (FRET) assay with an IC50 of 1.14 μM, but its modest potency was insufficient to modulate the production of IL-8 in IL-17A-stimulated human keratinocytes in the presence of TNF-α^23^,^24^.

To verify the specificity of compound **1** for IL-17A and the nature of its ability to disrupt IL-17 signaling, we used SPR to quantify its binding to the IL-17F homodimer. IL-17F was chosen because it has the highest sequence similarity to IL-17A (56% identity)^17^ in the IL-17 family of cytokines. Significantly, compound **1** did not show any measurable binding to the IL-17F homodimer at concentrations up to 40 μM. (Supplementary Fig. S1). Furthermore, compound **1** did not show measurable binding to the common receptor for IL-17 signaling, IL-17RA^14^,^15^,^18^, at concentrations up to 40 μM (Supplementary Fig. S1). Taking these results together, compound **1** is believed to inhibit the IL-17A/IL-17RA interaction via its specific and exclusive binding to the IL-17A cytokine.

In an effort to optimize this series, we undertook studies to understand both the druggability of IL-17A and the nature of its binding site for these compounds.

### Druggability assessment and molecular dynamics of IL-17A

**Figure 2:**
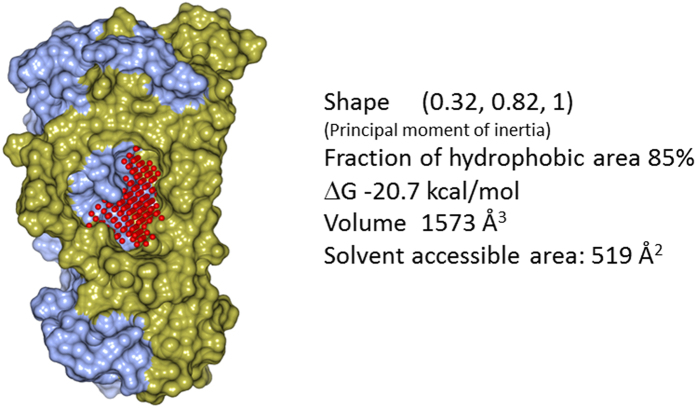
Characterization of the central binding pocket of the IL-17A dimer (surface presentation with the two polypeptide chains colored in ice blue and gold, respectively) probed using the VISM algorithm (red balls represent the probes used). The high druggability of the pocket is manifested by the large hydrophobic cavity and the favorable druggability score (∆G) which assesses the optimal binding affinity of the binding site.

The variational implicit solvent model algorithm (VISM)^25^ was applied to exhaustively probe the dimer surface of a published IL-17A structure^17^ for putative binding pockets. This study revealed a pocket in the center of the IL-17A dimer that appeared to be both highly flexible and druggable (Fig. 2) because its large volume permits that portion of the cytokine to switch between various conformational states. To gauge the potential of this pocket for small molecule modulation of IL-17A we assessed protein flexibility using molecular dynamics (MD) simulations. MD simulations of protein-ligand binary complexes with compound **1** docked in the central pocket revealed that ligand binding further stabilized the system under ambient conditions. A significant fraction of the different conformations available to the central pocket appeared druggable, qualifying this cavity as the starting point for a small-molecule discovery program.

### Determination of IL-17A/compound complex structures

**Figure 3:**
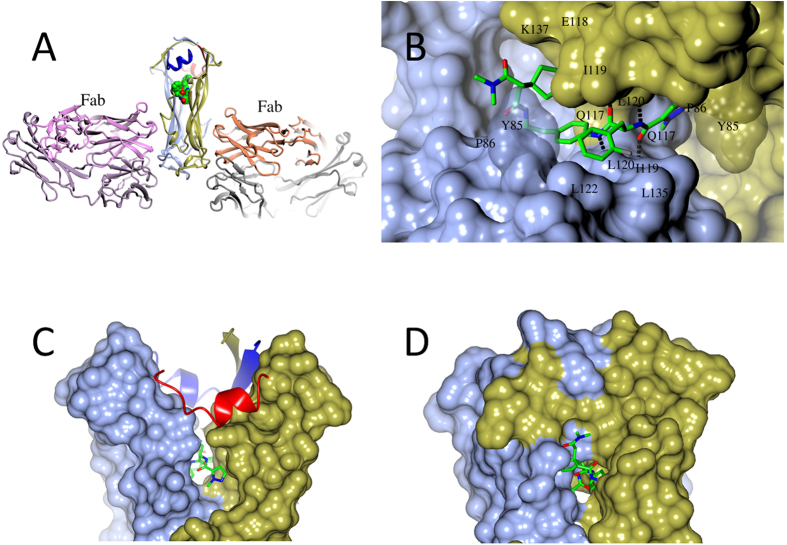
Binding of compound **1** in a central pocket of IL-17A. (**A**) Overall structure of the Fab/HAP/IL-17A/compound **1** complex. Component polypeptides appear as ribbons, with two HAP molecules in blue and red, respectively, and the two chains of IL-17A in ice blue and gold, respectively. Atoms of compound **1** appear as spheres, with carbons in green, nitrogens in blue, and oxygens in red. Hydrogen bonds are shown as dashes. Picture prepared using program CCP4MG^43^. (**B**) Close view of compound **1** bound in the central pocket at the IL-17A dimer interface and interacting with both IL-17A monomers (surface representation). Dashed lines are hydrogen bonds. (**C**) Compound **1** binding enlarges the central pocket and makes the N-terminal half of the IL-17A dimer much less compact. (**D**) For comparison, the central pocket is much smaller in the apo IL-17A dimer and completely enclosed. Compound **1** would clash with the closed binding site.

Protein structural information is especially valuable in drug discovery programs targeting PPIs. Unfortunately, in the present case, extensive co-crystallization and soaking of compounds from the lead series into preformed apo-IL-17A crystals^17^ failed to yield structures of protein-ligand complexes. Strong signals from thermal shift assays and extensive line broadening in NMR studies of binding made it evident that binding of these compounds to IL-17A resulted in significant conformational shifts of the protein relative to the apo structure (Supplementary Fig. S2). As we found previously that an anti-IL-17A Fab stabilized the complex of the cytokine with a high affinity peptide antagonist (HAP)^23^, and allowed its structure to be determined, we investigated whether the same approach would serve in the present case. Competitive binding studies by SPR showed not only that HAP failed to block compound **1** from binding to IL-17A but that it actually increased its on-rates (Supplementary Figure S3). As these compounds generally have shown slow to moderate on-rates of 10^2^–10^4 ^M^−1^s^−1^, we reasoned that HAP may stabilize a conformation of IL-17A that accommodates ligand binding and used both Fab and HAP to stabilize IL-17A in our subsequent crystallization efforts (Fig. 3). This strategy was successfully in enabling us to determine high resolution complex structures that revealed the binding mode of compound **1** (Supplementary Table 1). The observed ligand binding site was indeed the central pocket as predicted by MD simulations performed on the apo protein (Supplementary Figure S4).

### The IL-17A/compound 1 complex reveals a widened central pocket

The crystal structure of the complex of compound **1** with IL-17A reveals that binding occurs at the dimer interface (Fig. 3A) in the central pocket formed by residues from both IL-17A monomers (Fig. 3B). Compound binding dramatically reconfigures the dimer; the central pocket widens, loosening packing of the entire N-terminal half of the cytokine (Fig. 3C). In contrast, the apo IL-17A dimer is much more closed and has a smaller central pocket (Fig. 3D). Within the central pocket, residues Leu120 of each monomer (numbering is based on its precursor form) make hydrogen bonds with the amides of compound **1** (Fig. 3B). Upon ligand binding, the main chains of Leu120 move 3.4 Å away from each other relative to their positions in the apo structure, and this results in separation of the entire N-terminal β-strands of the two IL-17A polypeptides. The same movement eliminates multiple steric clashes of compound **1** with IL-17A and results in the IL-17A dimer interface area being decreased from 1666 Å^2^ in the apo structure to 1127 Å^2^ in the complex, and the number of interchain hydrogen bonds in the cytokine being reduced from 22 to 15. The calculated dimer binding energy was decreased by 10.6 kcal/mol, from −37.2 kcal/mol in the unbound structure to −26.6 kcal/mol in the liganded structure (IL-17A dimer interface parameters were calculated using program PISA^26^). The decrease in protein dimer binding energy indicates that this widened compound-bound conformation of IL-17A represents a higher energy state of the protein itself compared to its apo form. Such a large conformational change at the ligand binding site should pose a large energy barrier to conformational transition, and this expectation is confirmed by the observed low k_on_ values for compound **1** (Fig. 1) and other compounds in the series (data not shown). Achieving activity in this slow-binding series would require correspondingly slow off rates, and SPR studies showed that these were observed (Supplementary Fig. S5).

### The widened binding site of IL-17A complex is highly druggable

The druggability of the binding site revealed by the X-ray structure was also studied computationally using the VISM algorithm. Compared to apo IL-17A, both the solvent-accessible surface and the volume of the binding pocket are more than doubled upon binding of compound **1** (from 519 to 1316 Å^2^ and 1573 to 3362 Å^3^, respectively). Compound **1** covers 564 Å^2^ of hydrophobic surface area of the IL-17A dimer, and generates two hydrogen bonds to the polypeptide backbone at each Leu120 of the cytokine dimer (Supplementary Fig. S6). The nature of this pocket and our druggability calculations indicate that compounds related to **1** should be capable of high affinity interactions to this widened binding site. At this binding site, in addition to the hydrogen bonds mentioned, the central phenyl ring of compound **1** is deeply buried at the binding site and makes extensive hydrophobic interactions with the IL-17A dimer (Supplementary Fig. S6). The methylpyrazole and the central phenyl ring almost superimpose with each other after applying the 2-fold symmetry of the IL-17A dimer. The 2-fluorophenylalanine side chain makes hydrophobic contacts with Leu120, Leu122 and Leu135 of one monomer and the cyclopentyl group binds at a hydrophobic side pocket formed by atoms of Glu118, Ile119, Leu120 and Lys137 of the other monomer (Fig. 3B). The terminal amide of compound **1** is largely solvent exposed (Fig. 3 and Supplementary Fig. S6). Throughout the MD simulation, the hydrogen bonds between compound **1** and IL-17A were largely consistent and maintained. From a ligand binding perspective, the X-ray structure supports the SAR observed for compound **1**. Notably, attempts at modifying the two right-hand amides, alterations of the hydrophobic nature of either the central phenyl ring or the pendant cyclopentyl group, or inversion of the (*S*)-chirality of the 2-fluorophenylalanine (Fig. 1) resulted in a complete ablation of potency (data not shown).

### Orthogonal study of the compound binding mode using covalent probes

**Figure 4:**
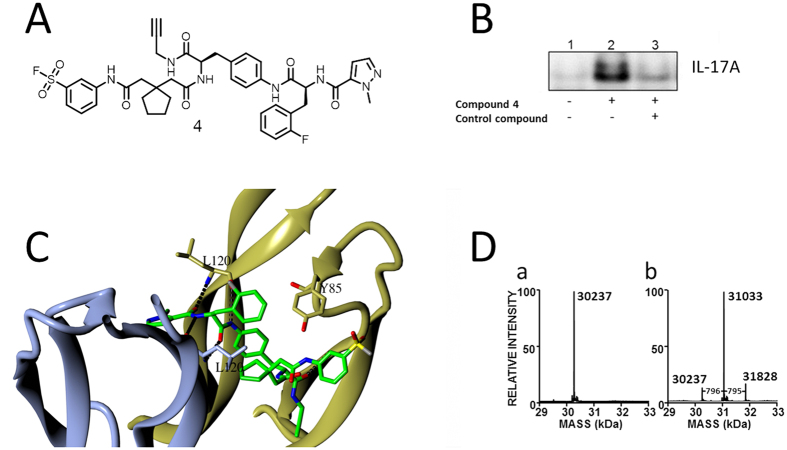
A covalent probe targeting the compound binding site in IL-17A. (**A**) Structure of compound **4,** an analog of compound **1** designed as a covalent probe of the IL-17A binding site with a FRET IC_50_ of 0.2 μM. (**B**) Compound **4** specifically and covalently labeled full length IL-17A. IL-17A labeled by compound **4** was biotinylated by click chemistry, and pulled down by streptavidin. Eluates were immunoblotted. (**C**) In this model of compound **4** in the IL-17A binding pocket, the reactive sulfonyl fluoride is close to Tyr85. (**D**) Mass spectra of IL-17A (a) before and (b) after treatment with compound **4**. The predicted mass shift resulting from a single modification of the protein was 795.3 Da.

Although the solved X-ray structure of IL-17A complexed with compound **1** was consistent both with MD analysis of the cytokine and the above SAR, we sought additional confidence in the relevance of the obtained binding mode to the protein in free solution. Several sulfonyl fluoride analogs of compound **1** were designed, prepared and evaluated for functional activity. The sulfonyl fluoride covalent warhead was the motif of choice owing to its role as a privileged electrophile that is known to react with a number of amino acid residues in a site-specific manner, including tyrosine^27^. Of the analogs surveyed, compound **4** (Fig. 4A) showed functional activity in our FRET assay (0.2 μM) and covalently labeled full-length recombinant IL-17A in the absence of HAP. Treatment of IL-17A with compound **4** unambiguously showed modification of the protein that was subject to competition by a non-covalent analog (Fig. 4B). Modeling predicted that the reactive sulfonyl fluoride of IL-17A-bound compound **4** would be positioned adjacent to Tyr85 of IL-17A (Fig. 4C).

Electrospray/time-of-flight mass spectrometry showed a major mass of 31,033 Da after treatment of the unmodified cytokine (30,237 Da) with the sulfonyl fluoride probe **4** (Fig. 4D). The 795.3 Da increase was consistent with one equivalent of compound **4** having modified the IL-17A dimer. Much smaller amounts of doubly labeled IL-17A were also detected, with the second modification attributed to nonspecific labeling (Fig. 4D). Peptide mapping of the modified protein confirmed Tyr85 as the principal site of modification by covalent probe **4,** consistent with our modeling and X-ray analysis (Supplementary Figs S7–S9).

### Structural basis for inhibition of the IL-17A/IL-17RA interaction by compound 1

**Figure 5:**
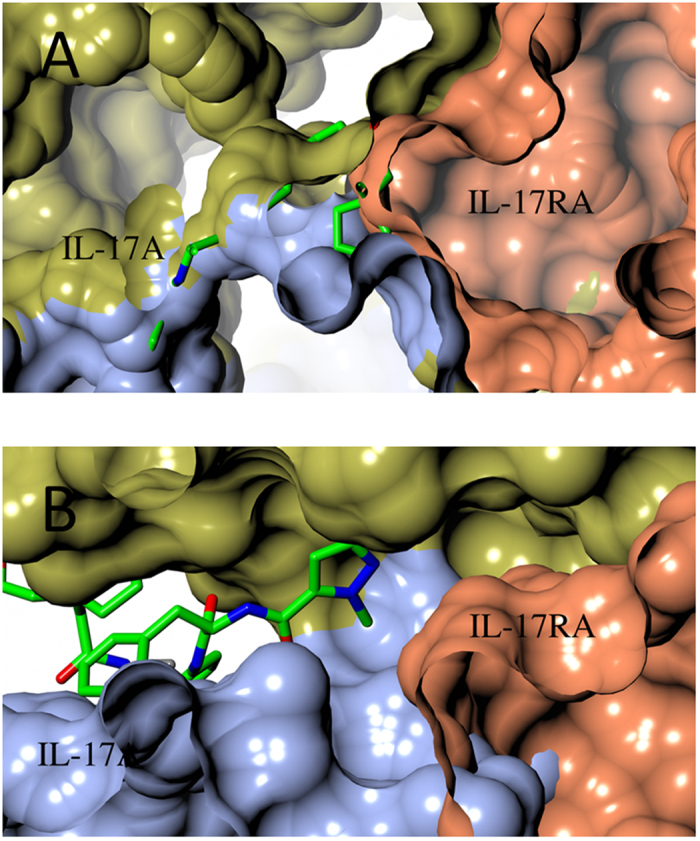
Mechanism of inhibition of the formation of the IL-17A/IL-17RA complex by compound **1**. (**A**) IL-17A/IL-17RA binary complex (PDB accession 4HSA) is incompatible with compound **1** binding. Compound **1** (stick model) was superimposed into its IL-17A binding site in IL-17A/IL-17RA complex (surface representation, with IL-17RA colored in coral). (**B**) IL-17A/compound **1** binary complex is incompatible with IL-17RA binding. IL-17RA (coral surface) was superimposed onto IL-17A/compound **1** binary complex.

Normal binding of dimeric IL-17A to IL-17RA is a 1:1 interaction between the proteins^15^,^17^ (Fig. 5), with the 2-fold symmetry of the cytokine allowing it to present either of two faces to the receptor. With the IL-17A/IL-17RA complex structure^17^ as a starting point, we modeled binding of the IL-17A/compound **1** binary complex to IL-17RA (Fig. 5). The cyclopentyl group of compound **1** clearly impedes binding of IL-17RA to one potential binding surface on the cytokine (Fig. 5A), but the other end of compound **1** is deeply buried in the cytokine and not capable of clashing with receptor binding on the cytokine’s other face (Fig. 5B). Nevertheless, the compound causes sufficient conformational changes in that region of IL-17A to perturb multiple cytokine-receptor interactions (Fig. 5B). The perturbations stem from disruption of hydrogen bonds and hydrophobic interactions previously shown to be important for the IL-17A/IL-17RA interaction^17^.

### The bound conformation of compound 1 suggests a macrocyclization strategy

**Figure 6:**
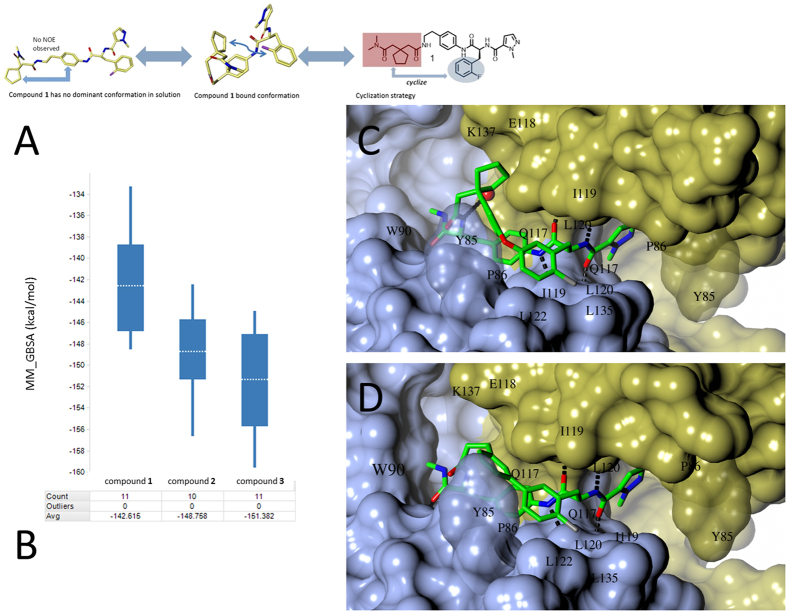
Cyclization strategy inspired by the bound bioactive conformation of compound **1**. (**A**) In solution, lack of an NOE signal from the cyclopentyl and phenyl linker of compound **1** (left) indicated that the bound conformation was not highly populated in solution. Cyclization between the L-phenylalanine side chain and the *N,N*-dimethylamide (right) may reinforce the bound bioactive conformation (right). (**B**) Use of MD/MM-GBSA to predict binding affinities in design of macrocyclic compounds. Examples are for the discussed compounds. Box regions correspond to 50% of the distribution, lines extend to max 1.5 times of this interval, and averages are denoted by dashed lines in the boxes. (**C**) Compound **2** bound to IL-17A. D. Compound **3** bound to IL-17A.

The crystal structure of the complex shows that compound **1** adopts a U-shaped conformation when bound to IL-17A (Fig. 6). In this conformation, the terminal dimethylamide is only 5.7 Ǻ away from the nearest atom in the ring of the 2-fluorophenylalanine residue. As compound **1** has 10 rotatable single bonds, it seemed unlikely that the bound conformation was the dominant one in solution. To examine the conformation of compound **1** in solution, a nuclear Overhauser effect (NOE) study was made of the compound dissolved in aqueous buffer. No NOE signal was detected that suggested adjacency of the cyclopentyl group to the central ring, suggesting that the bound conformation was not significantly populated in solution (Fig. 6A). The apparent divergence of the solution and bound conformations led us to postulate that the affinity of these compounds for the cytokine could be improved by using a macrocyclization strategy to lock in the bound conformation and thereby diminish the entropic costs of reorganization during binding (Fig. 6A)^28^.

### Macrocyclic compound design aided by affinity prediction using MD

Our design strategy was to construct a cyclization linker that connects the substituted phenylalanine side chain to the cyclopentyl-containing side chain (Fig. 6A). To evaluate a number of bond disconnections and avoid having to synthesize an excessive number of challenging macrocycles, we triaged potential analogs using MD simulations and molecular mechanics-generalized Born surface area (MM-GBSA) methods^29^. Binding affinities of the proposed structures were calculated. The design hypothesis was to favor the bound conformation of compound **1** while adapting to the observed binding site as much as possible, and then to enquire computationally whether improvements in affinity and functional activity would be realized. The free energy of binding was calculated by using a combination of gas phase energy (MM), electrostatic solvation energy (GB), and non-electrostatic contribution to solvation energy (SA). Combining this with MD simulations, the free energy of binding was estimated for ensembles of several ligand-protein conformations. The coupled MD/MM-GBSA approach was considered more likely to avoid the pitfall of failing to capture the energetically most favorable state from a static binary complex because it produces a distribution of possible binding events on the free energy landscape. For the proposed targets, we contrasted the calculated MD/MM-GBSA binding affinities with that calculated for compound **1** by the same methodology. Macrocycles that compared favorably with compound **1** were selected for synthesis (see Supplementary synthetic route). Two such proposed macrocycles, **2** and **3** (Fig. 1), did indeed compare favorably in terms of free energy of binding, as illustrated in the plot (Fig. 6B), and were prioritized. During MD simulation, the key interactions for compound **1** with IL-17A, including hydrogen bonds, are mostly maintained.

### Improvements of macrocyclic compound 2 and 3 over acyclic compound 1

Compound **2** and **3** each showed an improvement in affinity (SPR K_D_ < 200 nM) and functional activity (FRET IC_50_ < 35 nM). Importantly, they also exhibited potent activity in the psoriasis-relevant keratinocyte cellular assay measuring IL-17A-stimulated production of the pro-inflammatory cytokine IL-8 (IC_50_ < 540 nM) (Fig. 1). Moreover, macrocycle **3** achieved these improvements over compound **1** without a significant increase in molecular weight.

We also tested the specificities of macrocycle **2** and **3**. In SPR, while compounds **2** and **3** bound potently to IL-17A, they did not show measurable binding to IL-17F or IL-17RA at concentrations up to 13.3 μM (Supplementary Fig. S10). Furthermore, compound **2** and **3** did not inhibit the baseline IL-8 production of keratinocytes stimulated by TNF-α alone (IC_50 _> 30 μΜ) (Supplementary Fig. S11). These results showed that these macrocycles maintain their target specificity toward IL-17A both *in vitro* and in a cellular setting.

### Binding modes of compounds 2 and 3 to IL-17A

Separate X-ray structures of IL-17A complexed with macrocycles **2** and **3** were obtained in the same manner as for acyclic ligand **1** (Fig. 6C,D). The binding modes of all three compounds are quite similar, but compounds **2** and **3** place their lipophilic cyclization linkers proximal to the Leu120 and Tyr85 residues, creating the potential for additional van der Waals contacts that are not possible with compound **1**. Additionally, the *N*-methylamide incorporated into the macrocyclic linker makes an additional hydrogen bond to the backbone N-H of Trp90 (Fig. 6C,D). We postulate that enforcement of the bound conformation via macrocyclization and the introduction of new contacts made by the linker account for the increased activity of the macrocycles. The remaining interactions of compound **2** and **3** with IL-17A binding site are similar to those of compound **1** (Figs 3B vs 6C, D; Supplementary Fig. S6).

## Discussion

Crystallization of a complex between compound **1** and IL-17A and subsequent structural analysis showed that compound binding causes the highly flexible cytokine to adopt an open conformation in which a continuous cavity has formed between its two polypeptides. This open conformation was too distant from the free cytokine to be predictable in detail by MD, but it clearly is favored as part of the overall structure of the complex. The U-shaped conformation of compound **1** bound in the interchain cavity suggested that potency might be improved by macrocyclization, and the custom-designed macrocyclic compounds **2** and **3** were subsequently found to have improved affinity for IL-17A and activity in the FRET assay. Finally, as an even more distinct advance over compound **1**, they also demonstrated activity in the stimulated keratinocyte cellular assay.

No significant increase in on-rate was observed with macrocyclic compounds **2** and **3** as compared to acyclic compound **1**. The absence of any change suggested that protein conformational changes are rate determining for binding of these compounds and, consequently, the root cause of the slow on-rates. This is reasonable in view of the large conformational changes observed. Further support for this argument was the requirement to use “crystallization handlers”, the Fab and HAP components of the crystallized complexes, to assist in gaining co-crystals of compounds with the cytokine. The overall improvement in binding affinity (K_D_) for compounds **2** and **3** are the result of their significantly slower off-rates (Fig. 1).

The requirement for Fab and HAP “crystallization handlers” raises the question of whether the complex observed in the crystal structure is to any extent an artifact of the procedure required to obtain it, or is equally accessible to IL-17A treated with compound in solution. Treating the cytokine with the sulfonyl fluoride probe **4** resulted in a single modification of the dimeric IL-17A at Tyr85, a result that was both stoichiometrically and structurally consistent with the binding interaction derived from X-ray crystallography.

The structural observations and SPR profiles suggest that binding of this series of compounds to IL-17A requires more than a single step. It is more likely a multiple-step process that involves an induced fit. In this model, the ligand has an initial association with the cytokine to form a complex that is then rearranged to achieve the final binding conformation^30^,^31^. Under this mechanism, protein rearrangements have a large impact on the on-rate for the ligands. Notably, in the absence of HAP stabilization of the open conformation of the cytokine, the initial compound **1** complex structure with IL-17A could not be achieved (Supplementary Figure 3).

Compounds **1, 2** and **3** target the central pocket which is both important for IL-17RA binding and the weakest point in the IL-17A dimer interface due to the pre-existing cavity in the apo IL-17A structure (Fig. 2). The amino-acid sequences of regions that enclose the central pocket in IL-17A are not strictly conserved in IL-17 cytokines. For example, Ile119, which directly contacts the central phenyl ring of these compounds at the binding site of IL-17A (Figs 2B and 6C,D), is a hydrophilic Thr in IL-17F. Such a sequence difference explains the specificity of this series of compounds for IL-17A. We have clearly shown that these compounds bind specifically to IL-17A and not to IL-17F or the receptor IL-17RA. In the cell based assay, the improved potencies of compounds **2** and **3** allowed them to inhibit IL-17A-stimulated IL-8 production, but not the baseline production of IL-8 stimulated by TNF-α.

In conclusion, we have solved the X-ray structure of the complex of small molecule ligand **1** to IL-17A. Due to the flexible nature of this protein and the large conformational changes needed for ligand binding this could only be achieved with additional Fab and peptide stabilizers bound to the cytokine, but results obtained with a covalent reactive probe fully supported the relevance of the crystal structures to IL-17A in solution. Compound **1**, our initial lead derived from disclosures elsewhere, was active only in biochemical assays of cytokine-receptor interaction, and possessed so great a number of rotatable bonds that its U-shaped cytokine-bound conformation appeared unlikely to be significantly populated in free solution. This implies that binding to IL-17A would incur a substantial entropic penalty.

To reduce this, the structure was evolved by macrocyclization. Compounds **2** and **3** were first designed and then prioritized for synthesis, as MD simulations predicted that they would exhibit increased affinity for IL-17A. This expectation was duly realized, and the gain in potency associated with the new compounds was sufficient to yield measurable activity in the keratinocyte-based bioassay for IL-17A inhibition. Their increase in affinity for the target was largely attributable to reductions in their off-rates. The level of potency of this class of ligands makes them useful *in vitro* tools for studying the highly disease-relevant interaction of IL-17A with its principal receptor. They can also serve as the starting point for discovery of small molecule IL antagonists.

## Methods

### Primary human keratinocytes cell assay

Primary human keratinocytes were cultured in Epilife medium with EDGS (Life Technology, Cascade Biologics) following product instructions. 5 days after establishing the culture from frozen vials, cells were plated at 10,000/well (80 μL) in culture media in 384-well plates. 4 h after plating, 10 μL of 10× compound stocks were added. Final DMSO concentration was 1%. Immediately after compound addition, 10 μL of a mixture of recombinant human IL-17A (R&D System, Minneapolis, MN) (5 ng/mL final) and TNF-α (Sigma-Aldrich, St Louis, MO) (10 ng/mL final), or TNF-α alone (10 ng/mL) were added to the cells. Cell assay plates were incubated for 48 h at 37 °C in a tissue culture incubator. Culture supernatants were harvested for analysis of IL-8 production using kit K211ANB-2 (Meso Scale Discovery, Rockville, MD).

### SPR binding assays

The IL-17 SPR binding assay was run on a Biacore 3000 SPR instrument (GE Healthcare). Biotinylated Bap-tagged human IL-17A^23^, was captured on a Biacore Streptavidin chip to achieve a protein density of 2500–3500 RUs on the surface. The SPR running buffer was 10 mM HEPES, pH 7.4, 150 mM NaCl, 0.01% P20 with 3% DMSO. Compound samples were injected at a flow rate of 50 μL/min for 180 s association and followed by running buffer for at least 600 s to detect dissociation. Compound concentrations were varied across a 2–3 fold dilution series. Each concentration was tested at least in duplicate. When compounds were tested in the presence of HAP, the running buffer contained 50 nM of HAP and all samples were prepared in the running buffer. Multiple blank injections were run before and after each compound series for references. The data were processed and analyzed with Scrubber 2.0 (BioLogical Software) and Biaeval software (GE Healthcare) to calculate the binding constants along with on- and off-rates.

Biotinylated BAP-tagged IL-17RA ECD^17^ and chemically biotinylated IL-17F (R&D Systems, Minneapolis, MN) were separately immobilized on Biacore Streptavidin chips to permit selectivity testing of compounds. The binding experiments were carried out as described above.

### NMR

2D ^1^H-^13^C heteronuclear single quantum coherence (HSQC) correlation spectra were obtained with uniformly ^15^N, ^13^C labeled IL-17A expressed in *E. coli* (Supplementary Method). Samples containing 50  μM IL-17A and 250 μM compound from linear peptide series (with K_D_ = 0.1 μM) were prepared in 10 mM HEPES, pH 7.4, 150 mM NaCl containing 10% D_2_O. The DMSO concentration was maintained at 3%. NMR data was recorded on a Bruker Avance 600 MHz NMR spectrometer at 298 K. Spectra were acquired using 2048 × 128 complex points and spectral widths of 10,000 × 2300 Hz in t2 × t1 dimensions. The recycle delay was 1 s and 64 transients were recorded for each complex t1 point. All data were processed using TopSpin 2.1 (Bruker Instruments). The F1 dimension was zero filled to 256 points and sine function was applied to both dimensions prior to Fourier transform.

2D nuclear Overhauser effect spectroscopy (NOESY) spectra was recorded for compound **1** at 300 μM in 50 mM Tris-d, 150 mM NaCl in 100% D_2_O at pH 7.4 (not corrected for D_2_O) on a Bruker Avance 600MHz NMR spectrometer at 288 K and 298 K. Data was collected with 2048 complex points in the F2 dimension and 128 increments in the F1 dimension over a spectral width of 16 ppm and mixing times of 300 ms and 500 ms. The F1 dimension was zero filled to 512 and both dimensions were multiplied by a square – sine function prior to transformation.

### FRET Assay

The FRET signal of an Europium labeled IL-17A donor and an Alexa Fluor 647 labeled IL-17RA acceptor was measured to monitor the interaction of IL-17A and IL-17RA^23^. Maximal FRET was observed when IL-17A was bound to IL-17RA and diminished FRET was observed when IL-17A was separated from IL-17RA. Excitation of the donor at 320 nm triggers fluorescence at 615 nM and this in turn excites the acceptor, which fluoresces at 665 nm. Fluorescence was measured at both 615 nm and 665 nm and the 665/615 ratio was used to monitor IL-17A/IL-17RA binding. Final assay concentrations were 1 nM biotinylated IL-17A^23^ labeled with 0.67 nM Europium-Streptavidin (Invitrogen), 6 nM IL-17RA-Fc fusion protein (R&D Systems) labeled with 1 nM Alexa Fluor 647 antibody (BioLegend) in a buffer containing 10 mM HEPES pH 7.4, 150 mM NaCl, 0.02% BSA, and 0.01% Tween 20. Compounds were tested using a half-log dilution series of 11 concentrations. The IL-17A was incubated with the Europium-Streptavidin to 1.5X final assay concentration for 1 h at room temperature. Compounds were prepared at 50X final concentration in 100% DMSO and 300 nL were added to a 384-well white assay plate (Greiner). 10 μL of the Europium labeled IL-17A was added to the compounds and incubated at room temperature for 1 h. During this pre-incubation of compound and IL-17A, IL-17RA was incubated with Alexa Fluor 647 antibody to 3X final assay concentration at room temperature for one hour and then 5 μL of the 3X Alexa Fluor 647 labeled IL-17RA was added to the assay for a total volume of 15.3 μL and a final DMSO concentration of 2%. The plates were covered and incubated at room temperature for 3 h. The FRET signal of the IL-17A/IL-17RA interaction was measured using an EnVision Multilabel plate reader (PerkinElmer). The compound data was converted into % inhibition, using 0% (no HAP) and 100% inhibition (100 nM HAP) as controls. A four-parameter logistic nonlinear regression model using the percent inhibition at each concentration was used to calculate an IC_50_ for each compound.

### Protein production for crystallization

The full length IL-17A with signal peptide removed (residues 24–155, UniProtKB accession Q16552) was expressed in *E. coli* and refolded from inclusion bodies^23^,^32^. The refolded protein was purified by size exclusion chromatography and subjected to RP-HPLC for separation of the doubly disulfide-linked IL-17A homodimer from the dissociable non-covalent dimer^32^. The covalent linked IL-17A dimer was used in co-crystallization and compound **4** labeling studies. Existence of covalent linkage in IL-17A dimer used was confirmed by mass spectrometry and SDS polyacrylamide gel electrophoresis.

The anti-IL-17A antibody Fab fragment used for co-crystallization was designed based on the published crystal structure of an IL-17A/Fab complex^23^,^33^. A His6 tag was placed at the N-terminus of the heavy chain of the Fab to facilitate affinity purification. The Fab heavy and light chains were co-expressed in HEK293 cells (AkesoBio, China), and purified on a TALON metal affinity column, followed by size-exclusion on a Superdex 200 column. The Fab/IL-17A complex was generated by mixing one molar of IL-17A dimer with a slight excess of 2 molars of Fab, and separated from excess unbound Fab on a Superdex 200 column.

### Protein crystallization and data collection

To screen for crystallization conditions of Fab/full length IL-17A/HAP/compounds using commercial screen kits, 30 mM compounds and 30 mM HAP in DMSO stock were added at final concentrations of 1 mM each to the Fab/IL-17A complex at 8–10 mg/mL. Crystallization drops were set up in sitting drop plates, by mixing 200 nL of protein complex solution with 200 nL of well solution, and equilibrated against 80 μL of well solution at 20 °C. In the initial crystallization screens, a condition of 10% 2-propanol, 20–24% PEG 6K, 0.1 M sodium acetate pH = 4.0–5.0 consistently gave crystals. The condition was further optimized using a tool compound, 2-chloro-Nalpha-(phenylacetyl)-N-(4-{[(9R)-7,10,13,21-tetraoxo-8,11,14,20-tetraazaspiro[4.17]docos-9-yl]methyl}phenyl)phenylalaninamide, which routinely gave high diffracting crystals. To generate complex structures of different compounds described in this study, the crystals were transferred into 2 μL of cryo solution of 15% 2-propanol, 25% PEG 6K, 0.1 M sodium acetate pH = 4.4 and 25% glycerol, plus 1 mM compounds and 1 mM HAP, and equilibrated against 80 μL of cryo solution overnight. Crystals were harvested and directly flash cooled in liquid nitrogen before data collection. All crystal data sets were collected at APS IMCA 17ID beamline (Chicago, IL), and processed with autoPROC^34^ (Supplementary Table 1).

### Structure determination and refinement

Fab/IL-17A/HAP/compounds complex structures were solved by the molecular replacement method using the published Fab/IL-17A crystal structure as search model^33^ (pdb code 2VXS), using program Phaser^35^. Structure refinements were carried out using program Buster and manual model building using program COOT^36^.

### Binding site characterization by VISM

Using as a starting point the previously reported X-ray structure of IL-17A structure (PDB code: 4HSA), homology-based modeling was applied to build the missing loop connecting the N- and C- terminal domains to afford a symmetric IL-17A dimer. The VISM algorithm describes molecular solvation equilibrium by minimizing solvation free energy function with respect to the solute-solvent interface. This method differs from other implicit solvent models in that the solute–solvent interface is not predefined^37^, and it therefore, provides a physically more meaningful equilibrium solute-solvent interface as well as more accurate solvation free energy estimates. A retrospective study of binding pocket identification in a large number of protein-ligand crystallographic structures has shown good sensitivity and specificity^25^.

The VISM-CFA method was originally designed as an alternative implicit solvent model for molecular solvation^38^,^39^,^40^. Individual protein simulation frames were chosen as input structures. The partial charges and 12-6 Lennard-Jones (LJ) potential parameters of solute atoms are obtained from the Amber force field: TIP3P water Lennard-Jones (LJ) parameter = 0.152 kcal/mol; and the solvent molecular diameter = 3.15 Å. The macroscopic planar surface tension was set at γ_0_ = 0.076 kcal/mol/Å^2^ at 300 K which was obtained from the TIP3P water simulation. The Tolman coefficient τ was chosen to be 1 Å for the convex and concave atomic level surface tension correction. These parameters are consistent with the previous studies^40^,^41^.

### Molecular dynamics coupled with MM-GBSA

The ligand-protein binary complexes were generated by the Maestro molecular visualization suite and optimized by the protein preparation protocol (www.schrodinger.com). The molecular dynamics simulations were carried out with the Desmond module. First, the system was filled with explicit solvent molecules with a buffer distance of 10 Å in the orthorhombic simulation boxes. A relaxation protocol was applied which included minimization with and without constraints followed by a series of short molecular dynamics simulations using the Berendsen thermostat and Berendsen barostat. The production runs used 2 fs time steps with a duration of 20 ns for each ligand-protein complex. The OPLS_2005 force field was used with particle-mesh Ewald (PME) method for long range electrostatic interactions^42^. Trajectory analyses of MD simulations were done by extracting snapshots for the last 10 ns at 1 ns interval. The ten frames of the MD coordinates were subjected to affinity prediction by the molecular mechanics-generalized Born surface area (MM-GBSA) method^29^. The binding free energies (∆G in kcal/mol) were calculated using Prime module of the Schrodinger simulation suite: ΔG_bind_ = G_complex_ − G_protein_ − G_ligand_.

### Protein Mass Analysis

Human recombinant IL-17A expressed in *E. coli* (5 μM) was incubated with and without compound **4** (50 μM) for 20 h at 37 °C in Dulbecco’s phosphate-buffered saline solution. To analyze cytokine homodimer, 10 μL of each sample was subjected to LC-MS using an Acquity class H UPLC (Waters) interfaced to a Xevo G2–S Q-Tof mass spectrometer (Waters). Chromatography was performed on a 2.1 × 100 mm Acquity BEH 300 Å C4 column (1.7 μm silica) (Waters) at 40 °C using the following gradient: 5% B/1 min, 5–85% B/10 min at 0.4 mL/min. Solvents: A, 0.05% formic acid in water; B, 0.05% formic acid in acetonitrile. To analyze IL-17A peptide monomers, samples of reaction mixtures were treated with 4.5 mM DTT for 15 min at 22 °C before LC-MS was performed.

### LC-MS Peptide Mapping

100 μL of each reaction sample was treated with three volumes of acetone at −20 °C for 2 h, after which the mixture was centrifuged at 14,000 × *g* for 10 min. The supernatant was discarded, and dried precipitated protein was redissolved in 10 μL of 8 M urea, 0.2 M Tris-Cl, pH 7.5, and incubated for 15 min at 37 °C. The samples were diluted to 40 μL with water to 2 M urea, 0.05 M Tris-Cl, pH 7.5, and treated with 0.5 μg endoproteinase Lys-C (Wako) for 3 h at 37 ^o^C with the further addition of 0.5 μg trypsin (Promega) and continued incubation for 17 h at 37 °C. Each digest was next treated with 4.5 mM DTT for 15 min at 37 °C, and 8 μL of each digest was analyzed on an Agilent Model 1100 HPLC system interfaced with a LTQ Orbitrap XL mass spectrometer (Thermo). Peptides were fractionated by RP-HPLC on a 5 μm, 300 Å, 100 × 0.5 mm PROTO C18 column (Higgins) at 40 °C using the following gradient: 1.6% B/2 min, 1.6–35% B/98 min, 35–80% B/10 min at a flow rate of 10 μL/min. Solvents: A, 0.1% formic acid in water; B, 0.1% formic acid in acetonitrile. Mass spectra from *m/z* 400–2000 were acquired in the Orbitrap, with the most abundant ions targeted for concurrent MS/MS in the linear ion trap using a collision energy of 35%, a 2 Da isolation width, and dynamic exclusion set to 3 with an exclusion time of 30 s.

### IL-17A labeling with compound 4

0.5 μM full length IL-17A (R&D Systems) was pre-incubated with DMSO or 50 μM a control compound in PBS at room temperature for 30 min, after which 5 μM sulfonyl fluoride probe compound **4** was added to the protein mixture (final sample volume 50 μl) and incubated for 1 h. Click chemistry was then performed at room temperature for 2 h. Briefly, 38.2 μL of 4% SDS in HEPES buffer (pH 7.5) was added to the sample, followed by the addition of 1.9 μL of 4 mM biotin azide (Life Technologies), 2 μL of 50 mM CuSO_4_, 5.9 μL TBTA in DMSO:*tert*-BuOH (1:5), and 2 μL of 50 mM TCEP (final concentrations: 1.5% SDS, 75 μM biotin azide, 1 mM CuSO_4_, 5% *tert*-BuOH and 1 mM TCEP). 1 mL of 6 M urea in PBS was added to quench the reaction. The mixture was then incubated with 100 μL high-capacity streptavidin agarose (Thermo Fisher Scientific) at room temperature for 2 h with rotation. Beads were centrifuged at 1000 g for 1 minute and washed 3 times with 1 mL 4 M urea in PBS. Proteins were eluted by adding 90 μL of 2× LDS sample buffer (Life Technologies) and heated at 95 °C for 10 minutes. Eluates were separated on a 12% NuPAGE Bis-Tris gel, transferred to a PVDF membrane, and then analyzed by immunoblot (anti-IL17A antibody from R&D Systems).

## Additional Information

**How to cite this article**: Liu, S. *et al*. Binding site elucidation and structure guided design of macrocyclic IL-17A antagonists. *Sci. Rep.* **6**, 30859; doi: 10.1038/srep30859 (2016).

## Supplementary Material

Supplementary Information
