# Supplementary material for: Binding site elucidation and structure guided design of macrocyclic IL-17A antagonists
Source: Sci Rep. 2016 Aug 16;6:30859. doi: 10.1038/srep30859 (PMC4985813; doi:10.1038/srep30859)
Supplement: Supplementary Information [file srep30859-s1.pdf]

Supplementary Material

Binding site elucidation and structure guided design of macrocyclic IL-17A antagonists

Shenping Liu, Leslie A. Dakin, Li Xing, Jane M. Withka, Parag V. Sahasrabudhe, Wei Li, Mary Ellen Banker, Paul Balbo, Suman Shanker, Boris A. Chrunyk, Zuojun Guo, Jinshan M. Chen, Jennifer A. Young, Guoyun Bai, Jeremy T. Star, Stephen W. Wright, Joerg Bussenius, Sheng Tan, Ariamala Gopalsamy, Bruce A. Lefker, Fabien Vincent, Lyn H. Jones, Hua Xu, Lise Hoth, Kieran F. Geoghegan, Xiayang Qiu, Mark E. Bunnage and Atli Thorarensen

Supplementary compounds synthesis routes and ^{1}H NMR characterizations

Supplementary method

Supplementary Table S1

Supplementary Figure S1-11

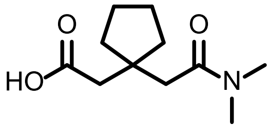

2-(1-(2-(dimethylamino)-2-oxoethyl)cyclopentyl)acetic acid: Et_3N (72 g, 713 mmol) was added to a suspension of dimethylamine hydrochloride (29 g, 357 mmol) in toluene (100 mL). The suspension was stirred for 10 min at 15 °C, after which 8-oxaspiro[4.5]decane-7,9-dione (30 g, 178 mmol) was added. The mixture was heated under reflux for 12 h, then concentrated. The residue was taken up in EtOAc (2 L) and washed with 1 M HCl (2 × 500 mL), brine (1 L), dried with anhydrous Na_2SO_4, filtered and concentrated *in vacuo* to afford the title compound (33.5 g, 88%) as a brown oil which was used without further purification. ^{1}H NMR (400 MHz, CDCl3) δ = 3.16 (s, 3H), 3.05 (s, 3H), 2.58 (s, 2H), 2.49 (s, 2H), 1.84 - 1.62 (m, 6H), 1.52 - 1.43 (m, 2H)

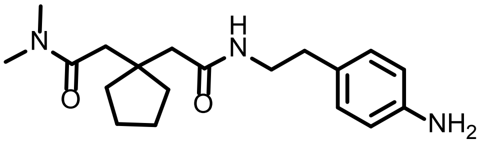

2-(1-(2-((4-aminophenethyl)amino)-2-oxoethyl)cyclopentyl)-*N*,*N*-dimethylacetamide: To a stirred solution of 2-(1-(2-(dimethylamino)-2-oxoethyl)cyclopentyl)acetic acid (26.9 g, 126 mmol) in DMF (150 mL) was added 4-(2-aminoethyl)aniline (27.7 g, 132 mmol), EDCI (26.6 g, 139 mmol) and HOBT (18.7 g, 139 mmol) with stirring at 18 °C, followed by the addition of DIEA (48.9 g, 378 mmol). The solution was stirred at 18 °C for 30 h before being diluted with EtOAc (1 L). The EtOAc was separated, washed with aqueous $NaHCO_3$ solution (2 × 500 mL), water (1 L), brine (2 L), dried with anhydrous Na_2SO_4, filtered and concentrated *in vacuo* to afford the title compound (22 g, 52%) as a brown oil which was used without further purification. ^{1}H NMR (400 MHz, CDCl3) δ = 7.40 (br. s., 1H), 6.99 (d, J=8.5 Hz, 2H), 6.61 (d, J=8.0 Hz, 2H), 3.57 (br. s., 2H), 3.48 - 3.39 (m, 2H), 3.05 (s, 3H), 2.95 (s, 3H), 2.69 (t, J=7.5 Hz, 2H), 2.38 (s, 2H), 2.36 (s, 2H), 1.78 - 1.58 (m, 6H), 1.48 - 1.35 (m, 2H) LCMS: 331 (M + H+), 354 (M + Na+)

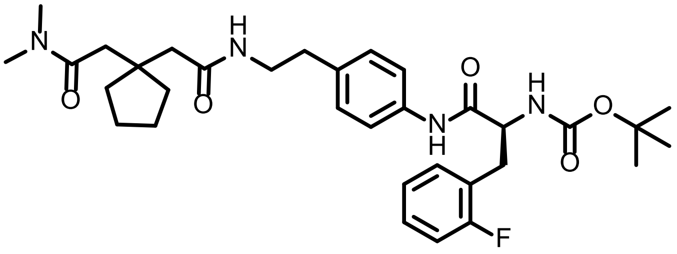

***tert*-butyl-(*S*)-(1-((4-(2-(2-(1-(2-(dimethylamino)-2-oxoethyl)cyclopentyl)acetamido)ethyl)phenyl)amino)-3-(2-fluorophenyl)-1-oxopropan-2-yl)carbamate**: A solution of **2-(1-(2-((4-aminophenethyl)amino)-2-oxoethyl)cyclopentyl)-*N*,*N*-dimethylacetamide** (20 g, 60 mmol), (*S*)-2-((*tert*-butoxycarbonyl)amino)-3-(2-fluorophenyl)propanoic acid (18.8 g, 66.4 mmol) and DIEA (19.5 g, 151 mmol) in DCM (200 mL) was treated with a solution of propanephosphonic anhydride (50% in EtOAc, 57.6 g, 90 mmol). The solution was stirred at 25 °C for 4 h before being quenched with aqueous $NaHCO_3$. The organic layer was separated and the aqueous layer was extracted with additional DCM (2 × 200 mL). The combined DCM extracts were washed with brine (250 mL), dried with anhydrous Na_2SO_4, filtered and concentrated *in vacuo* to afford crude title compound (28 g) as a yellow solid. This material was triturated with MTBE to afford the title compound (27 g, 75%) as an off-white solid. ^1H NMR (400 MHz, $CDCl_3$) δ = 7.87 (br. s., 1H), 7.58 (br. s., 1H), 7.34 (d, J=8.5 Hz, 2H), 7.30 - 7.22 (m, 2H), 7.15 (d, J=8.5 Hz, 2H), 7.12 - 7.03 (m, 2H), 5.11 (br. s., 1H), 4.52 - 4.41 (m, 1H), 3.52 - 3.45 (m, 2H), 3.04 (s, 3H), 2.95 (s, 3H), 2.78 (t, J=7.0 Hz, 2H), 2.38 (s, 2H), 2.30 (s, 2H), 1.76 - 1.60 (m, 6H), 1.40 (s, 9H), 1.39 - 1.34 (m, 2H), LCMS: 597 (M + H+)

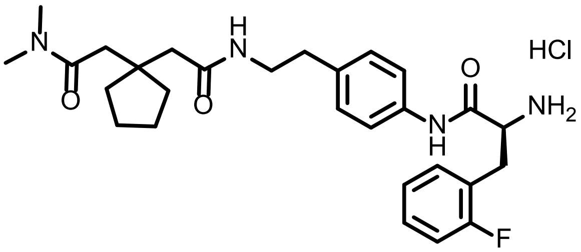

(*S*)-2-amino-*N*-(4-(2-(2-(1-(2-(dimethylamino)-2-oxoethyl)cyclopentyl)acetamido)ethyl)phenyl)-3-(2-fluorophenyl)propanamide hydr°Chloride: To a solution of *tert*-butyl-(*S*)-(1-((4-(2-(2-(1-(2-(dimethylamino)-2-oxoethyl)cyclopentyl)acetamido)ethyl)phenyl)amino)-3-(2-fluorophenyl)-1-oxopropan-2-yl)carbamate (27 g, 45 mmol) in dioxane (200 mL) was added 4 M HCl in dioxane (200 mL).

The solution was stirred at 18 °C for 16 h before the solvent was removed *in vacuo*. The residue was triturated with MTBE and filtered. The filter cake was washed with MTBE (2 × 50 mL) and dried to afford the title compound (22.3 g, 93%) as a yellow solid. ^{1}H NMR (400 MHz, D_2O) δ = 7.34 - 7.26 (m, 1H), 7.26 - 7.19 (m, 1H), 7.19 - 7.13 (m, 2H), 7.13 - 7.04 (m, 4H), 4.25 (dd, J=5.8, 8.3 Hz, 1H), 3.44 (t, J=6.3 Hz, 2H), 2.90 (s, 3H), 2.81 (s, 3H), 2.75 (t, J=6.3 Hz, 2H), 2.17 (br. s., 2H), 2.16 (s, 2H), 1.51 - 1.39 (m, 4H), 1.33 - 1.21 (m, 4H), LCMS: 497 (M + H+), HPLC: 95.3% purity

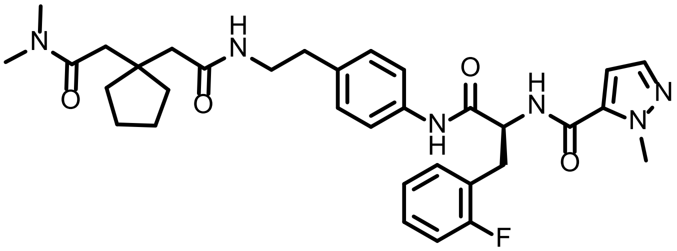

Compound 1: (*S*)-*N*-(1-((4-(2-(2-(1-(2-(dimethylamino)-2-oxoethyl)cyclopentyl)acetamido)ethyl)phenyl)amino)-3-(2-fluorophenyl)-1-oxopropan-2-yl)-1-methyl-1H-pyrazole-5-carboxamide (1): A solution of **(*S*)-2-amino-*N*-(4-(2-(2-(1-(2-(dimethylamino)-2-oxoethyl)cyclopentyl)acetamido)ethyl)phenyl)-3-(2-fluorophenyl)propanamide hydrochloride** (600 mg, 1.2 mmol), 1-methyl-1H-pyrazole-5-carboxylic acid (183 mg, 1.4 mmol) and DIEA (390 mg, 3.0 mmol) in DCM (25 mL) was treated with a solution of propanephosphonic anhydride (50% in EtOAc, 1150 mg, 1.8 mmol). The mixture was stirred at 25 °C for 12 h before being diluted with DCM (20 mL). The solution was washed with $NaHCO_3$ (20 mL), brine (20 mL), dried with anhydrous Na_2SO_4, filtered and concentrated *in vacuo*. The residue was purified chromatography on silica gel (0% to 10% MeOH in DCM) to afford the title compound (200 mg) as a yellow solid. This was dissolved in MeOH (5 mL) and water (10 mL). The suspension was evaporated and the residue was lyophilized overnight to provide the title compound (158 mg, 22%) as a white solid. ^{1}H NMR (400 MHz, DMSO) δ = 10.14 (s, 1H), 8.79 (d, J=8.0 Hz, 1H), 7.88 (t, J=5.3 Hz, 1H), 7.50 (d, J=8.5 Hz, 2H), 7.44 (d, J=2.0 Hz, 1H), 7.39 (t, J=7.3 Hz, 1H), 7.28 - 7.19 (m, 1H), 7.17 - 7.04 (m, 4H), 6.96 (d, J=2.0 Hz, 1H), 4.93 - 4.83 (m, 1H), 3.95 (s, 3H), 3.27 - 3.20 (m, 2H), 2.92 (s,

3H), 2.78 (s, 3H), 2.64 (t, J=7.0 Hz, 2H), 2.44 (s, 2H), 2.23 (s, 2H), 1.58 - 1.48 (m, 6H), 1.48 - 1.39 (m, 2H), LCMS: 605 (M + H+), HPLC: 98.9% purity

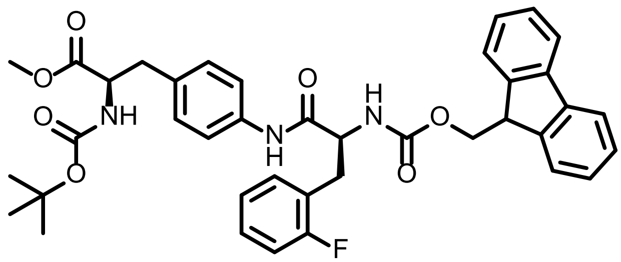

Methyl-(*R*)-3-(4-((S)-2-((((9H-fluoren-9-yl)methoxy)carbonyl)amino)-3-(2-fluorophenyl)propanamido)phenyl)-2-((*tert*-butoxycarbonyl)amino)propanoate: A solution of (*S*)-2-((((9H-fluoren-9-yl)methoxy)-carbonyl)amino)-3-(2-fluorophenyl)propanoic acid (300 mg, 0.74 mmol) in DMF (5 mL) was treated with HATU (309 mg, 0.81 mmol) and *N*-methylmorpholine (90 mg, 0.89 mmol). The mixture was stirred at 20 °C for 10 min before the addition of a solution of methyl (*R*)-3-(4-aminophenyl)-2-((tert-butoxycarbonyl)amino)propanoate (240 mg, 0.81 mmol) in DMF (1 mL). The solution was stirred at 20 °C for 18 h before being diluted with water (20 mL). The mixture was extracted with EtOAc (2 × 30 mL) and the combined organic extract was washed with H_2O (2 × 30 mL), dried with anhydrous Na_2SO_4, filtered and concentrated *in vacuo* to afford the title compound (563 mg, >100%) as a light yellow solid, which was estimated to be 80% pure and was used without further purification. ^{1}H NMR (400 MHz, $CDCl_3$) δ = 7.85 (br. s., 1H), 7.76 (d, J=7.5 Hz, 2H), 7.52 (d, J=7.0 Hz, 2H), 7.44 - 7.33 (m, 4H), 7.33 - 7.21 (m, 4H), 7.13 - 7.02 (m, 4H), 5.49 (br. s., 1H), 4.97 (d, J=8.5 Hz, 1H), 4.61 - 4.49 (m, 2H), 4.45 - 4.37 (m, 1H), 4.36 - 4.28 (m, 1H), 4.18 (t, J=7.0 Hz, 1H), 3.71 (s, 3H), 3.30 - 3.14 (m, 2H), 3.13 - 2.98 (m, 2H), 1.43 (s, 9H), LCMS: 704 (M + Na+)

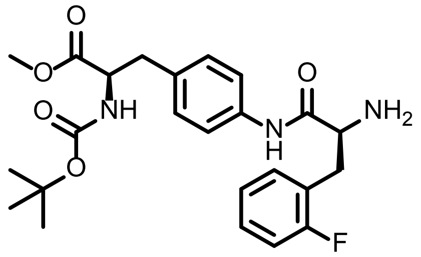

Methyl-(*R*)-3-(4-((S)-2-amino-3-(2-fluorophenyl)propanamido)phenyl)-2-((tert-butoxycarbonyl)amino)-propanoate: Piperidine (4.00 g, 47 mmol) was added to a solution of unpurified methyl(*R*)-3-(4-((S)-2-((((9H-fluoren-9-yl)methoxy)carbonyl)amino)-3-(2-fluorophenyl)propanamido)phenyl)-2-((*tert*-butoxycarbonyl)amino)propanoate (2.00 g, 2.35 mmol, in DCM (30 mL). The solution was stirred at 18 °C for 4 h before being concentrated to dryness. The residue was purified by chromatography on silica gel (0% to 4% MeOH in DCM) to afford the title compound (768 mg, 71%) as yellow gum. ^{1}H NMR (400 MHz, $CDCl_3$) δ = 9.46 (br. s, 1H), 7.53 (d, J=8.0 Hz, 2H), 7.33 - 7.20 (m, 2H), 7.16 - 7.03 (m, 4H), 4.98 (d, J=8.0 Hz, 1H), 4.63 - 4.51 (m, J=7.5 Hz, 1H), 3.75 (dd, J=4.0, 9.5 Hz, 1H), 3.72 (s, 3H), 3.43 (dd, J=3.8, 13.8 Hz, 1H), 3.14 - 2.99 (m, 2H), 2.90 (dd, J=9.5, 13.6 Hz, 1H), 1.43 (s, 9H), LCMS: 482 (M + Na+)

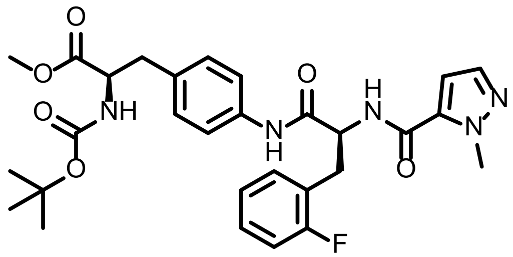

Methyl-(*R*)-2-((*tert*-butoxycarbonyl)amino)-3-(4-((*S*)-3-(2-fluorophenyl)-2-(1-methyl-1H-pyrazole-5-carboxamido)propanamido)phenyl)propanoate: A solution of 1-methyl-1H-pyrazole-5-carboxylic acid (171 mg, 1.36 mmol) in DMF (10 mL) was treated with HATU (517 mg, 1.36 mmol) and *N*-methylmorpholine (250 mg, 2.47 mmol). The mixture was stirred at 20 °C for 10 min before the addition of a solution of **methyl-(*R*)-3-(4-((*S*)-2-amino-3-(2-fluorophenyl)propanamido)phenyl)-2-((tert-butoxycarbonyl)amino)-propanoate** (568 mg, 1.236 mmol) in DMF (5 mL). The solution was stirred at 20 °C for 18 h before being diluted with water (10 mL) and extracted with EtOAc (2 × 30 mL). The extracts were dried (Na_2SO_4), filtered and concentrated *in vacuo* to afford the title compound (1.10 g, >100%) as yellow oil, which was used without further purification in the next step. ^{1}H NMR (400 MHz, CDCl3) δ = 8.21 (br. s, 1H), 7.59 - 7.53 (m, 2H), 7.43 (d, J=2.0 Hz, 1H), 7.38 (d, J=8.5 Hz, 2H), 7.13 - 7.02 (m, 4H), 6.89 (d, J=8.0 Hz, 1H), 6.54 (d, J=2.5 Hz, 1H), 4.98 (d, J=8.5 Hz, 1H), 4.60 - 4.51 (m,

J=7.0 Hz, 1H), 4.17 - 4.13 (m, 1H), 4.12 (s, 3H), 3.71 (s, 3H), 3.34 - 3.24 (m, 2H), 3.11 - 2.99 (m, J=5.5, 14.1 Hz, 2H), 1.42 (s, 9H), LCMS: 590 (M + Na+).

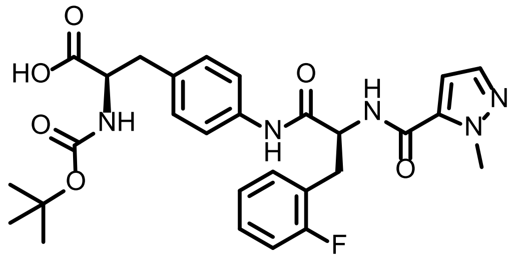

(*R*)-2-((*tert*-butoxycarbonyl)amino)-3-(4-((*S*)-3-(2-fluorophenyl)-2-(1-methyl-1H-pyrazole-5-carboxamido)propanamido)phenyl)propanoic acid: NaOH (186 mg, 4.65 mmol) was added to a solution of methyl(*R*)-2-((*tert*-butoxycarbonyl)amino)-3-(4-((*S*)-3-(2-fluorophenyl)-2-(1-methyl-1H-pyrazole-5-carboxamido)propanamido)phenyl)propanoate (1.10 g, 1.36 mmol theory) in MeOH (18 mL) and H_2O (2 mL) at 20 °C. The mixture was stirred at 20 °C for 6 h before being concentrated to dryness. The residue was dissolved in H_2O (2 mL) and extracted with EtOAc (2 × 10 mL). These extracts were discarded. The aqueous phase was cooled to 0 °C and adjusted to pH 5 with 1 N HCl. A white suspension formed which was extracted with EtOAc (2 × 40 mL). The extracts were dried with anhydrous Na_2SO_4, filtered and concentrated *in vacuo* to afford the title compound (830 mg, 97%) as a white solid. ^{1}H NMR (400 MHz, DMSO) δ = 10.09 (br. s, 1H), 8.75 (d, J=8.0 Hz, 1H), 7.47 (d, J=8.5 Hz, 2H), 7.44 (d, J=2.0 Hz, 1H), 7.38 (t, J=6.8 Hz, 1H), 7.27 - 7.19 (m, 1H), 7.19 - 7.12 (m, 3H), 7.11 - 7.04 (m, 1H), 6.93 (d, J=2.0 Hz, 1H), 4.92 - 4.83 (m, 1H), 4.06 - 3.97 (m, 1H), 3.95 (s, 3H), 3.26 - 3.17 (m, 1H), 3.11 - 3.02 (m, 1H), 2.95 (s, 1H), 2.82 - 2.71 (m, 1H), 1.32 (s, 9H), LCMS: 576 (M + Na+), HPLC: 94.2% purity.

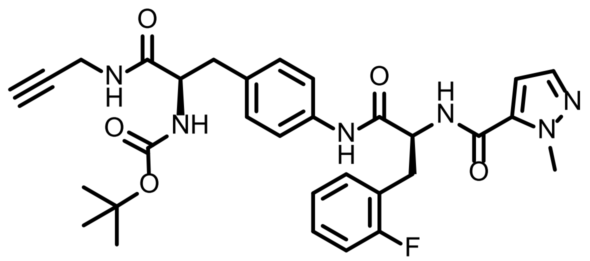

***tert*-butyl((*R*)-3-(4-((*S*)-3-(2-fluorophenyl)-2-(1-methyl-1H-pyrazole-5-carboxamido)propanamido)-phenyl)-1-oxo-1-(prop-2-yn-1-ylamino)propan-2-yl)carbamate**: A solution of (*R*)-2-((*tert*-butoxycarbonyl)amino)-3-(4-((*S*)-3-(2-fluorophenyl)-2-(1-methyl-1H-pyrazole-5-carboxamido)propanamido)phenyl)propanoic acid (500 mg, 0.90 mmol) in DMF (10 mL) was treated with HATU (378 mg, 0.99 mmol) and DIPEA (233 mg, 1.81 mmol). The mixture was stirred at 20 °C for 10 min before the addition of a solution of propargylamine (55 mg, 0.99 mmol) in DMF (1 mL). The solution was stirred at 20 °C for 16 h before being diluted with water (10 mL). The mixture was extracted with EtOAc (2 × 30 mL). The extracts were washed with H_2O (10 mL), brine (20 mL), dried (Na_2SO_4), filtered and concentrated to afford the title compound (518 mg, 97%) as a light yellow solid, which was used without further purification in the next step. ^1H NMR (400 MHz, DMSO) δ = 4.09 (br. s., 1H), 8.73 (d, J=8.0 Hz, 1H), 8.38 (t, J=5.5 Hz, 1H), 7.47 (d, J=8.5 Hz, 2H), 7.44 (d, J=1.0 Hz, 1H), 7.38 (t, J=7.5 Hz, 1H), 7.27 - 7.20 (m, 1H), 7.17 (d, J=8.0 Hz, 2H), 7.14 - 7.04 (m, 2H), 6.93 (d, J=1.0 Hz, 1H), 6.90 (d, J=8.5 Hz, 1H), 4.92 - 4.82 (m, J=7.0 Hz, 1H), 4.13 - 4.04 (m, 1H), 3.95 (s, 3H), 3.86 (d, J=4.0 Hz, 2H), 3.22 (dd, J=5.5, 13.1 Hz, 1H), 3.12 (s, 1H), 3.06 (dd, J=9.8, 13.8 Hz, 1H), 2.87 - 2.80 (m, 1H), 2.68 - 2.62 (m, 1H), 1.30 (s, 9H) LCMS: 613 (M + Na+)

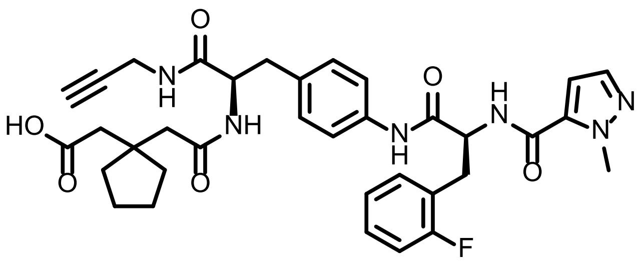

2-(1-(2-(((*R*)-3-(4-((*S*)-3-(2-fluorophenyl)-2-(1-methyl-1H-pyrazole-5-carboxamido)propanamido)phenyl)-1-oxo-1-(prop-2-yn-1-ylamino)propan-2-yl)amino)-2-oxoethyl)cyclopentyl)acetic acid: TFA (2 mL) was added to a solution of *tert*-butyl((*R*)-3-(4-

((*S*)-3-(2-fluorophenyl)-2-(1-methyl-1H-pyrazole-5-carboxamido)propanamido)-phenyl)-1-oxo-1-(prop-2-yn-1-ylamino)propan-2-yl)carbamate (200 mg, 0.30 mmol) in DCM (10 mL) at 0 °C. The solution was stirred at 20 °C for 3 h, before being concentrated to dryness. The resulting oil was dissolved in anhydrous THF (5 mL) and treated with Et_3N (131 mg, 1.3 mmol) for 10 min at 20 °C, after which 8-oxaspiro[4.5]decane-7,9-dione (65 mg, 0.39 mmol) was added. The solution was stirred at 60 °C for 16 h before being filtered and concentrated to dryness. The residue was purified by chromatography on silica gel (0% to 8% MeOH in DCM). Fractions containing the desired product were pooled and concentrated *in vacuo*, then dissolved in DCM (20 mL) and washed with brine (2 × 10 mL) and H_2O (10 mL). The DCM solution was dried (Na_2SO_4), filtered and concentrated *in vacuo* to afford the title compound (180 mg, 85%) as a yellow solid. ^{1}H NMR (400 MHz, DMSO) δ = 10.06 - 10.03 (m, J=6.0 Hz, 1H), 8.71 (d, J=8.5 Hz, 1H), 8.40 (t, J=5.5 Hz, 1H), 8.05 (d, J=9.0 Hz, 1H), 7.46 (d, J=8.5 Hz, 2H), 7.44 (d, J=2.0 Hz, 1H), 7.40 - 7.34 (m, 1H), 7.27 - 7.21 (m, 1H), 7.15 (d, J=8.0 Hz, 2H), 7.13 - 7.05 (m, 2H), 6.93 (d, J=2.0 Hz, 1H), 5.75 (s, 1H), 4.92 - 4.82 (m, 1H), 4.51 - 4.41 (m, 1H), 3.95 (s, 3H), 3.85 (t, J=5.8 Hz, 2H), 3.22 (dd, J=4.8, 13.8 Hz, 1H), 3.10 (br. s., 1H), 3.08 - 2.99 (m, J=14.1 Hz, 1H), 2.97 - 2.88 (m, 1H), 2.73 - 2.63 (m, 1H), 2.31 - 2.21 (m, 2H), 2.21 - 2.13 (m, 2H), 1.53 - 1.37 (m, 6H), 1.28 - 1.19 (m, 2H), LCMS: 659 (M + H+), 681 (M + Na+)

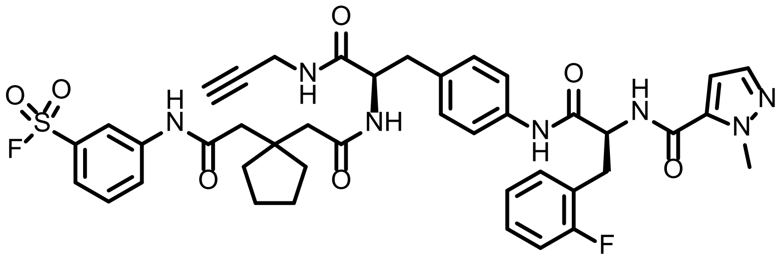

Compound 4: 3-(2-(1-(2-(((*R*)-3-(4-((S)-3-(2-fluorophenyl)-2-(1-methyl-1H-pyrazole-5-carboxamido)propanamido)-phenyl)-1-oxo-1-(prop-2-yn-1-ylamino)propan-2-yl)amino)-2-oxoethyl)cyclopentyl)acetamido)benzene-sulfonyl fluoride: A solution of 2-(1-(2-(((*R*)-3-(4-((*S*)-3-(2-fluorophenyl)-2-(1-methyl-1H-pyrazole-5-carboxamido)propanamido)phenyl)-1-oxo-1-(prop-2-yn-1-ylamino)propan-2-yl)amino)-2-oxoethyl)cyclopentyl)acetic acid (160 mg, 0.24 mmol) and 3-aminobenzenesulfonyl fluoride (51 mg, 0.29 mmol) in pyridine (1.2 mL) was cooled to 0 °C and treated with P°Cl_3 (45 mg, 0.29 mmol). The solution was stirred at 15 °C for 2 h, after which another portion of P°Cl_3 (45 mg, 0.29 mmol) was added. The solution was

stirred at 15 °C for an additional 3 h before being diluted with H_2O (5 mL). The mixture was extracted with EtOAc (2 × 10 mL). The extracts were washed with 1 M HCl (2 × 5 mL), brine (5 mL), dried with with anhydrous Na_2SO_4, filtered and concentrated *in vacuo*. The residue was purified by preparative HPLC and lyophilized to afford the title compound (10 mg, 5%) as a white solid. ^{1}H NMR (400 MHz, DMSO) δ = 10.65 (br. s, 1H), 10.07 (br. s, 1H), 8.74 (d, J=8.0 Hz, 1H), 8.55 (s, 1H), 8.47 (t, J=4.5 Hz, 1H), 8.21 (d, J=8.0 Hz, 1H), 7.88 (d, J=7.5 Hz, 1H), 7.79 - 7.66 (m, 2H), 7.51 - 7.41 (m, 3H), 7.37 (t, J=7.5 Hz, 1H), 7.28 - 7.20 (m, J=6.5 Hz, 1H), 7.17 (d, J=8.0 Hz, 2H), 7.13 - 7.03 (m, 2H), 6.93 (s, 1H), 4.92 - 4.81 (m, J=6.0 Hz, 1H), 4.57 - 4.46 (m, 1H), 3.94 (s, 3H), 3.86 (s, 2H), 3.25 - 3.13 (m, 2H), 3.11 (s, 1H), 3.09 - 3.02 (m, 1H), 2.95 (dd, J=3.3, 13.3 Hz, 1H), 2.74 - 2.63 (m, 1H), 2.30 (t, J=12.8 Hz, 2H), 2.20 (d, J=13.6 Hz, 1H), 1.58 - 1.39 (m, 6H), 1.38 - 1.20 (m, 2H), LCMS: 816 (M + H+), HPLC: 96.3% purity

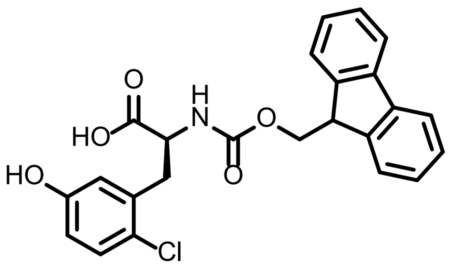

(*S*)-3-(2-Chloro-5-hydroxy-phenyl)-2-(9H-fluoren-9-ylmethoxycarbonylamino)-propionic acid: To a solution of (*S*)-3-(5-hydroxy-phenyl)-2-(9H-fluoren-9-ylmethoxycarbonylamino)-propionic acid (3.00 g, 7.44 mmol) in DMF (30 mL) was added a solution of N-chlorosuccinimide (0.99 g, 7.44 mmol) in DMF (20 mL) at 0 °C over 20 min. The resulting reaction mixture was stirred at 15 °C for 16 h. EtOAc (100 mL) was added, and the mixture was washed with water (3 × 250 mL), and brine (20 mL), dried over anhydrous Na_2SO_4, and concentrated *in vacuo* to afford the crude product (3.3 g) as a yellow oil.

The crude material were combined with 15.8 g crude material from 6 other batches obtained as above (from additional 14.50 g, 35.96 mmol starting material) to provide a total of 19.1 g crude material, which was purified by prep. HPLC at 220 nm (Phenomenex Luna C18, 10 μm, 250 x 80 mm, water/0.1%TFA-acetonitrile, gradient from 20% to 40% in 3 min, then 40% to 64% in 24 min, 64% to 95% in 1 min, hold at 95% for 5 min, back to 20% in 0.5 min, hold at 20% for

9.5 min, flow rate 150 mL/min, 19 injections). The clean product containing fractions were combined and concentrated to remove the acetonitrile, and the remaining aqueous layer was extracted with EtOAc. The combined EtOAc layers were dried over Na_2SO_4, and concentrated to afford the title compound (9.40 g, 49% yield, based on total 17.50 g starting material) as a white solid. An analytical sample (300 mg) was lyophilized from acetonitrile (5 mL) and water (20 mL). TLC (CH_2Cl_2:MeOH, 10:1 v/v): R_f= 0.16; $[\alpha]_D^{27}$= -24.8 (c = 0.13, methanol); ^1H NMR (400 MHz, MeOD): δ 7.79 (d, J = 7.5 Hz, 2H), 7.62 (dd, J = 3.8, 7.3 Hz, 2H), 7.51 (d, J = 8.5 Hz, 1H), 7.43 - 7.36 (m, 2H), 7.31 (q, J = 7.3 Hz, 2H), 7.18 (d, J = 8.8 Hz, 1H), 6.81 (d, J = 3.0 Hz, 1H), 6.67 (dd, J = 2.9, 8.7 Hz, 1H), 4.61 - 4.51 (m, 1H), 4.34 - 4.24 (m, 1H), 4.24 - 4.16 (m, 2H), 3.41 - 3.35 (m, 1H), 2.97 (dd, J = 10.0, 13.8 Hz, 1H); ^{13}C NMR (125 MHz, MeOD): δ 175.1, 158.5, 157.5, 145.2, 142.5, 137.2, 131.1, 128.7, 128.2, 126.4, 126.3, 125.2, 120.9, 119.6, 116.4, 68.2, 55.0, 36.6; Analytical chiral SFC (Chiralpak AD-3, 3 μm, 100 × 4.6mm, mobile phase: A: CO_2 B:ethanol (0.05% DEA), gradient: from 5% to 40% of B in 4.5 min and hold 40% for 2.5 min, then 5% of B for 1 min, flow rate: 2.8mL/min, column temperature: 40 $^{℃}$): t_R = 4.08 min, 99.8%; (Chiralpak AS-3, 3 μm, 100 × 4.6mm, mobile phase: A: CO_2 B: ethanol (0.05% DEA), gradient: from 5% to 40% of B in 4.5 min and hold 40% for 2.5 min, then 5% of B for 1 min, flow rate: 2.8 mL/min, column temperature: 40 $^{℃}$): t_R = 3.05 min, 100%; Analytical HPLC at 220 nm (Ultimate XB-C18, 3 μm, 50 x 3.0 mm, gradient 1% ACN in water (0.1% TFA) to 5% ACN in water (0.1% TFA) in 1 min; then from 5% ACN in water (0.1% TFA) to 100% ACN (0.1% TFA) in 5 min, hold at 100% ACN (0.1% TFA) for 2 min), back to 1% ACN in water (0.1% TFA) at 8.01 min, and hold 2 min, flow rate:1.2 mL/min): t_R = 4.75 min, 96%; HRMS (*m/z*): $[MH]^+$ cal'd. for $C_{24}H_{21}ClNO_5$, 438.1103; found, 438.1120.

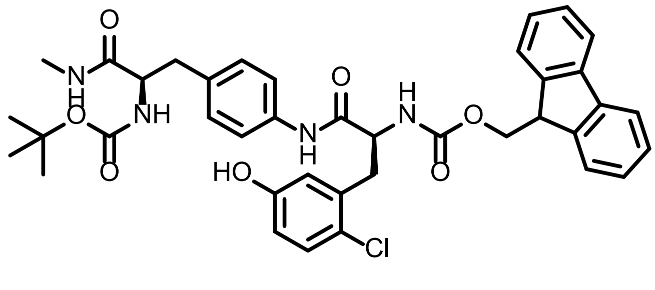

(*R*)-(2-{4-[(*S*)-3-(2-Chloro-5-hydroxy-phenyl)-2-(9H-fluoren-9-ylmethoxycarbonylamino)-propionylamino]-phenyl}-1-methylcarbamoyl-ethyl)-carbamic acid *tert*-butyl ester: To a solution of (*S*)-3-(2-Chloro-5-hydroxy-phenyl)-2-(9H-fluoren-9ylmethoxycarbonyl- amino)--propionic acid (3.30 g, 7.54 mmol) in DMF (60 mL) was added HATU (3.01 g, 7.91 mmol) and DIPEA (1.58 mL, 9.04 mmol) at 0 °C, and the resulting mixture was stirred at 0 °C for 10 min. Then (*R*)-[2-(4-amino-phenyl)-1-methylcarbamoyl-ethyl]-carbamic acid *tert*-butyl ester (2.05 g, 7.39 mmol, Klopfenstein, S.R. US 20040167183) was added, the mixture was warmed to 20 °C, and then stirred at this temperature for 16 h. The solution was diluted with EtOAc (200 mL), washed with brine (3 × 150 mL), the organic layer was dried over anhydrous Na_2SO_4 and concentrated *in vacuo* to give the crude title compound (5.5 g, ~65% purity) as a yellow solid, which was used directly for the next step. An analytical sample of 300 mg was purified by prep. HPLC (Phenomenex Gemini C18, 10 μm, 250 x 50 mm, water (0.22% formic acid) - acetonitrile, gradient from 47% to 87% over 10 min, flow rate 30 mL/min, 4 injections), and lyophilized to provide the title compound (94 mg) as a colorless solid. TLC (CH_2Cl_2:MeOH, 10:1 v/v): R_f= 0.26; $[\alpha]_D^{27}$= +2.2 (c = 0.13, DMF); ^{1}H NMR (400 MHz, DMSO-d_6): δ 9.91 (s, 1H), 9.58 (s, 1H), 7.88 (d, *J* = 7.3 Hz, 2H), 7.84 - 7.75 (m, 2H), 7.69 (t, *J* = 8.2 Hz, 2H), 7.47 (d, *J* = 8.0 Hz, 2H), 7.41 (t, *J* = 7.4 Hz, 2H), 7.30 (t, *J* = 7.5 Hz, 2H), 7.15 (d, *J* = 8.3 Hz, 2H), 7.19 (d, *J* = 8.5 Hz, 1H), 6.90 - 6.80 (m, 2H), 6.65 (dd, *J* = 2.8, 8.8 Hz, 1H), 4.46 (d, *J* = 6.0 Hz, 1H), 4.30 - 4.14 (m, 3H), 4.06 (d, *J* = 4.8 Hz, 1H), 3.12 - 2.83 (m, 3H), 2.74 - 2.63 (m, 1H), 2.58 (d, *J* = 4.5 Hz, 3H), 1.31 (s, 9H); Analytical chiral SFC (Chiralpak AD-H, 5 μm, 250 × 4.6mm, mobile phase: A: CO_2 B:ethanol (0.05% DEA), gradient: from 5% to 40% of B in 5 min and hold 40% for 2.5 min, then 5% of B for 2.5 min, flow rate: 2.5 mL/min, column temperature: 35 $^{°C}$): t_R = 5.69 min, 100%; (Chiralcel OJ-3, 3 μm, 150 × 4.6mm, mobile phase: A: CO_2 B:ethanol (0.05% DEA), gradient: from 5% to 40% of B in 5 min and hold 40% for 2.5 min, then 5% of B for 2.5 min, flow rate: 2.5 mL/min, column temperature: 35 $^{°C}$): t_R = 4.05 min, 100%; Analytical HPLC at 220 nm (Ultimate XB-C18, 3 μm, 50 x 3.0 mm, gradient 1% to 5% ACN in water (0.1% TFA) in 1 min; then from 5% to 100% ACN in 5 min, hold at 100% ACN for 2 min, flow rate: 1.2 mL/min): t_R = 5.09 min, 99%; HRMS (*m/z*): $[MH]^+$ cal'd. for $C_{39}H_{42}ClN_4O_7$, 713.2737; found, 713.2736.

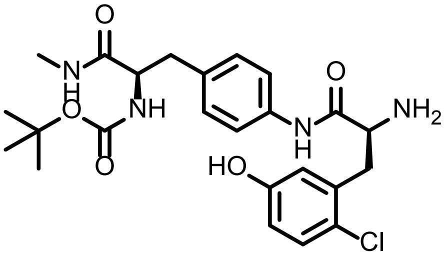

(*R*)-(2-{4-[(*S*)-2-Amino-3-(2-chloro-5-hydroxy-phenyl)-propionylamino]-phenyl}-1-methyl-carbamoylethyl)-carbamic acid *tert*-butyl ester: To a solution of piperidine (10 mL) in DMF (60 mL) was added (*R*)-(2-{4-[(*S*)-3-(2-Chloro-5-hydroxy-phenyl)-2-(9H-fluoren-9-ylmethoxycarbonylamino)propionylamino]-phenyl}-1-methylcarbamoyl-ethyl)-carbamic acid *tert*-butyl ester (4.50 g, 4.07 mmol, 65% pure), and the resulting light yellow solution was stirred at 20 °C for 16 h. EtOAc (200 mL) was then added and the organic phase was washed with water (3 × 200 mL), dried over anhydrous Na_2SO_4, and concentrated *in vacuo*. Purification by column chromatography (silica gel, 40 g, MeOH/CH_2Cl_2 = 0 to 10 %) afforded the title compound (1.52 g, 76% yield) as a yellow solid. An analytical sample of 120 mg was purified by prep. HPLC (DuraShell 5 µm, 150 x 25 mm, water (0.05% ammonium hydroxide) - acetonitrile, gradient from 27% to 47% over 10 min, flow rate 30 mL/min, 2 injections), and lyophilized to provide pure title compound (104 mg) as a colorless solid. TLC (CH_2Cl_2:MeOH, 10:1 v/v): R_f= 0.22; $[\alpha]_D^{27}$= +16.5 (c = 0.14, DMF); ^{1}H NMR (400 MHz, DMSO-d_6): δ 9.79 (br. s., 1H), 9.54 (br. s., 1H), 7.80 (d, *J* = 4.5 Hz, 1H), 7.49 (d, *J* = 8.3 Hz, 2H), 7.25 - 7.05 (m, 3H), 6.84 (d, *J* = 8.3 Hz, 1H), 6.79 (d, *J* = 3.0 Hz, 1H), 6.63 (dd, *J* = 2.9, 8.7 Hz, 1H), 4.10 - 3.99 (m, 1H), 3.60 - 3.52 (m, 1H), 3.03 (dd, *J* = 5.8, 13.6 Hz, 1H), 2.87 (dd, *J* = 4.8, 13.8 Hz, 1H), 2.77 - 2.63 (m, 2H), 2.57 (d, *J* = 4.5 Hz, 3H), 1.93 (br. s., 2H), 1.31 (s, 9H); ^{13}C NMR (100 MHz, DMSO-d_6): δ 173.5, 172.4, 156.5, 155.7, 137.6 , 137.5, 133.6, 130.1, 129.7, 123.3, 119.6, 118.8, 115.5, 78.4, 56.3, 56.2, 39.0, 37.6, 28.6, 26.0; Analytical chiral SFC (Chiralcel OD-3, 3 µm, 150 × 4.6 mm, mobile phase: A: CO_2 B:ethanol (0.05% DEA), gradient: from 5% to 40% of B in 5 min and hold 40% for 2.5 min, then 5% of B for 1 min, flow rate: 2.5 mL/min, column temperature: 35 °C): t_R = 4.07 min, 100%; Analytical HPLC at 220 nm (Ultimate XB-C18, 3 µm, 50 x 3.0 mm, gradient 1% to 5% ACN in water with 0.1% TFA in 1 min; then from 5% to 100% ACN in 5 min, hold at 100% ACN for 2 min, flow rate:1.2 mL/min): t_R = 3.37 min, 98%; HRMS (*m/z*): $[MH]^+$ cal'd. for $C_{34}H_{32}ClN_4O_5$, 491.2056; found, 491.2050.

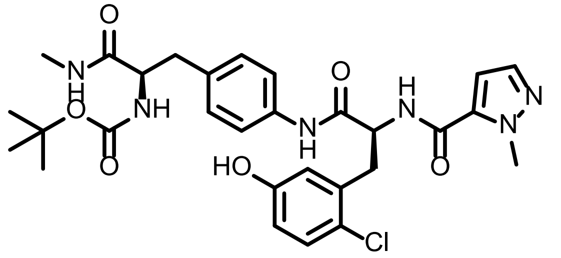

(*R*)-[2-(4-{(*S*)-3-(2-Chloro-5-hydroxy-phenyl)-2-[(2-methyl-2H-pyrazole-3-carbonyl)-amino]propionylamino}-phenyl)-1-methylcarbamoyl-ethyl]-carbamic acid *tert*-butyl ester: To a solution of 1-methyl-1H-pyrazole-5-carboxylic acid (0.40 g, 3.14 mmol) in DMF (60 mL) was added HATU (1.14 g, 2.99 mmol) and DIPEA (1.09 mL, 6.27 mmol) at 0 °C, and the resulting mixture was stirred at 0 °C for 10 min. (*R*)-(2-{4-[(*S*)-2-Amino-3-(2-chloro-5-hydroxy-phenyl)-propionylamino]-phenyl}-1-methylcarbamoylethyl)-carbamic acid *tert*-butyl ester (1.40 g, 2.85 mmol) was then added, the mixture was warmed to 20 °C and the reaction was allowed to stir at this temperature for 16 h. The solution was diluted with EtOAc (200 mL), washed with brine (3 × 150 mL), the organic layer was dried over anhydrous Na_2SO_4 and concentrated *in vacuo* to afford the crude product (1.5 g) as a brown gum. This material was combined with 0.11 g from another batch obtained as above and purified by column chromatography (silica gel, 40 g, $MeOH/CH_2Cl_2$ = 0 to 10%) to give the titel compound (1.50 g, 88% yield) as a grey solid. Further purification by SFC (Chiralcel OD, 5 μm, 250 × 30 mm, mobile phase: A: CO_2 B: ethanol, isocratic 25% B, flow rate: 60 mL/min) afforded a yellow solid compound **XIV** (550 mg, 32% yield). An analytical sample of 100 mg was additionally purified by prep. HPLC (Phenomenex Gemini C18, 5 μm, 150 x 25 mm, water (0.22% HCOOH) - acetonitrile, gradient from 25% to 55% over 10 min, flow rate 30 mL/min, 3 injections), and lyophilized to provide **XIV** (50 mg) as a white solid. TLC (CH_2Cl_2:MeOH, 10:1 v/v): R_f= 0.23; $[\alpha]_D^{26}$= +24.4 (c = 0.15, DMF); ^{1}H NMR (400 MHz, DMSO-d$_6$): δ 10.08 - 9.95 (m, 1H), 9.60 - 9.51 (m, 1H), 8.82 - 8.66 (m, 1H), 7.90 - 7.76 (m, 1H), 7.54 - 7.42 (m, 3H), 7.21 - 7.11 (m, 3H), 6.99 - 6.93 (m, 1H), 6.90 - 6.85 (m, 1H), 6.84 - 6.81 (m, 1H), 6.66 - 6.59 (m, 1H), 4.92 - 4.82 (m, 1H), 4.12 - 4.02 (m, 1H), 3.99 (s, 3H), 3.25 - 3.14 (m, 1H), 3.12 - 3.02 (m, 1H), 2.94 - 2.81 (m, 1H), 2.73 - 2.63 (m, 1H), 2.61 - 2.55 (m, 3H), 1.31 (s, 9H); ^{13}C NMR (100 MHz, DMSO-d$_6$): δ 171.9, 169.3, 159.3, 156.0, 155.1, 137.1, 136.8, 135.9, 134.8, 133.4, 129.6, 129.2, 122.8, 119.5, 117.8, 115.2, 107.8,

77.9, 55.8, 53.0, 38.8, 34.9, 28.1, 25.5; Analytical chiral SFC (Chiralcel OD-3, 3 μm, 150 × 4.6 mm, mobile phase: A: CO_2 B:ethanol (0.05% DEA), gradient: from 5% to 40% of B in 5 min and hold 40% for 2.5 min, then 5% of B for 2.5 min, flow rate: 2.5 mL/min, column temperature: 35 ℃): t_R = 4.54 min, 100%; Analytical HPLC at 220 nm (Ultimate XB-C18, 3 μm, 50 x 3.0 mm; gradient of 1% to 5% ACN in water (0.1%TFA) in 1 min, 5% to 100% ACN in 5 min, hold at 100% ACN for 2 min; flow low rate: 1.2 mL/min): t_R = 3.99 min, 99%; HRMS (*m/z*): [M+2H-B°C]$^+$ cal'd. for $C_{24}H_{28}ClN_6O_4$, 499.1861; found, 499.1895.

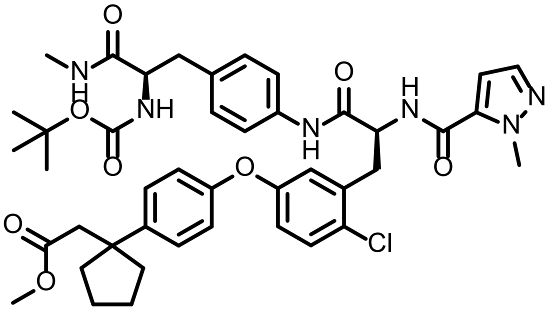

Methyl-2-(1-(4-(3-((S)-3-((4-((*R*)-2-((*tert*-butoxycarbonyl)amino)-3-(methylamino)-3-oxopropyl)-phenyl)amino)-2-(1-methyl-1H-pyrazole-5-carboxamido)-3-oxopropyl)-4-chlorophenoxy)phenyl)cyclopentyl)acetate: A round bottom flask equipped with a magnetic stirrer was added molecular sieves (4 A, 600 mg). The flask was then kept under vacuum and heated with a heat gun for 5 min. (*R*)-[2-(4-{(*S*)-3-(2-Chloro-5-hydroxy-phenyl)-2-[(2-methyl-2H-pyrazole-3-carbonyl)-amino]propionylamino}-phenyl)-1-methylcarbamoyl-ethyl]-carbamic acid *tert*-butyl ester was then added (150 mg, 0.25 mmol), $Cu(OAc)_2$ (57 mg, 0.31 mmol) and (4-(1-(2-methoxy-2-oxoethyl)cyclopentyl)phenyl)boronic acid (83 mg, 0.38 mmol) were then added followed by DMF (1 mL) and DCM (10 mL). The resulting mixture was then allowed to stir at ambient temperature for 20 h. Triethylamine (88 uL, 1.25 mmol) was then added and mixture was stirred at ambient temperature for an additional 24 h. LCMS indicated about 5% conversion to desired product by ELSD. The mixture was then stirred at ambient temperature for 10 days. LCMS suggested conversion to the desired product to be 10%. Additional $Cu(OAc)_2$ (38 mg, 0.25 mmol), (4-(1-(2-methoxy-2-oxoethyl)- cyclopentyl)phenyl)boronic acid (44 mg, 0.2 mmol) and molecular sieves (600 mg) were then added and the mixture was stirred in the microwave at 80 °C for 1 h and then ambient temperature for 20 h. The solvent was then removed *in vauco*

and the resulting residue was diluted with 5 mL DCM. The desired product was purified via on a silica gel chromatography (4 g) that was eluted with 30% to 100% EtOAc in heptane over 20 column volumes at a flow rate of 12 mL/min. ^{1}H NMR (400 MHz, DMSO-d_6) δ 10.01 (s, 1H), 8.62 (d, J = 8.3 Hz, 1H), 7.88 (s, 2H), 7.72 (m, 2H), 7.49 (d, J = 8.3 Hz, 2H), 7.34 (d, J = Hz, 1H), 7.33 (m, 2H), 7.07 (m, 3H), 6.89 (d, J = 2 Hz, 1H), 6.72 (m, 3H), 6.57 (d, J = 8.3 Hz, 1H), 4.94 (m, 1H), 4.11 (m, 1H), 3.90 (s, 3H), 3.34 (s, 3H), 3.30 (m, 3H with water peak), 3.08 (dd, J = 14.1, 9.4 Hz, 1H), 2.92 (m, 1H), 2.77 (m, 1H), 2.72 (m, 1H), 2.60 (m, 1H), 2.53 (d, J = 4.6 Hz, 3H), 1.93 (m, 2H), 1.80 (m, 2H), 1.61 (m, 2H), 1.51 (m, 2H), 1.18 (s, 9H). LCMS (*m/z*): $[M-H]^+$ cal'd for $C_{43}H_{51}ClN_6O_8$ 813.35; found,813.89.

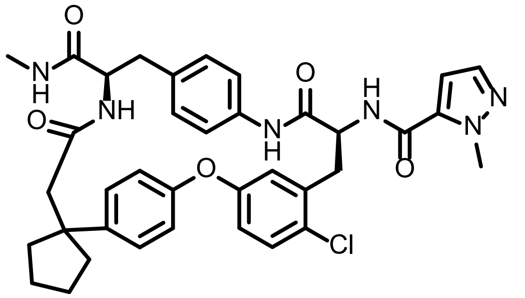

Compound 2: (5'*S*,10'*R*)-4'-chloro-5'-((1-methyl-1H-pyrazole-5-carbonyl)-l2-azanyl)-10'-((methyl-l2-azanyl)carbonyl)-7'l2,11'l2-spiro[cyclopentane-1,14'-2-oxa-7,11-diaza-1,8(1,4),3(1,3)-tribenzenacyclotetradecaphane]-6',12'-dione. To a round-bottom flask charged with methyl-2-(1-(4-(3-((S)-3-((4-((*R*)-2-((*tert*-butoxycarbonyl)amino)-3-(methylamino)-3-oxopropyl)-phenyl)amino)-2-(1-methyl-1H-pyrazole-5-carboxamido)-3-oxopropyl)-4-chlorophenoxy)phenyl)cyclopentyl)acetate (22 mg, 0.027 mmol) was added 1 mL THF, 1 mL DMF and LiOH (10 equiv., 0.27 mmol, MW 24, 6.5 mg) and 0.10 mL H_2O. The resulting mixture was stirred at ambient temperature for 1.5 h and then at 70 °C for 4 h before being allowed to cool to ambient temperature. The reaction flask was then placed in a refrigerator for 20 h. The solvent was then concentrated *in vacuo* and the resulting residue then dissolved in 1 mL dimethylformamide and 1 mL THF. To the resulting solution was added 2 mL 4 N HCl dioxane solution and the mixture was then stirred at RT for 2.5 h and concentrated *in vacuo*. To the round-bottom flask containing the saponified and des-Boc material (2.6 mg, 0.027 mmol) was added 0.5 mL DMF and DIPEA (3.3 μL, 0.14 mmol) followed by HATU (2.1 mg, 0.040 mmol). The reactions was stirred at ambient temperature for 15 min, LCMS confirmed

product formation and reaction completion. The reaction mixture was directly loaded on a 5 g C18 column which was eluted with 0 to 70% acetonitrile in water over 17 column volumes and then 70% to 100% acetonitrile over 23 column volume at 12 mL/min. Pure fractions were combined and concentrated to furnish the title compound as a white solid (0.61 mg, 24%). ^{1}H NMR (600 MHz, DMSO-d_6, two sets of peaks with a ratio of 1:1, only one set reported) δ 10.00 (s, 1H), 8.83 (d, *J* = 6.7 Hz, 1H), 7.79 (d, *J* = 8.6 Hz, 1H), 7.72 (q, *J* = 4.6 Hz, 1H), 7.51 – 7.49 (m, 2H), 7.42 (m, 2H), 7.17 (d, *J* = 8.5 Hz, 2H), 7.08 (d, *J* = 2.1 Hz, 1H), 7.00 (dd, *J* = 8.7, 2.9 Hz, 1H), 6.93 (d, *J* = 2.9 Hz, 1H), 6.63 (d, *J* = 8.9 Hz, 2H), 6.43 (d, *J* = 8.9 Hz, 2H), 4.71 (m, 1H), 4.29 (ddd, *J* = 11.5, 8.7, 2.5 Hz, 1H), 4.05 (s, 3H), 3.27 – 3.16 (m, 2H), 2.92 (dd, *J* = 13.6, 2.0 Hz, 1H), 2.67 (dd, *J* = 13.6, 11.7 Hz, 1H), 2.59 (d, *J* = 4.6 Hz , 3H), 2.53 (d, *J* = 14.1 Hz, 1H), 2.22 (d, *J* = 14.1 Hz, 1H), 2.13 – 2.01 (m, 2H), 1.95 – 1.90 (m, 2H), 1.73 (m, 2H), 1.66 – 1.58 (m, 2H). ^{13}C NMR (150 MHz, DMSO-d_6, two sets of peaks with a ratio of 1:1, only one set reported) δ 172.4, 170.8, 169.4, 159.6, 154.6, 154.1, 142.4, 137.6, 137.3, 136.5, 135.1, 134.1, 131.6, 129.9, 129.9, 128.6, 127.4, 127.4, 124.4, 120.8, 119.6, 119.6, 116.2, 116.2, 108.3, 54.9, 54.4, 48.5, 46.5, 40.8, 39.4, 37.7, 37.4, 36.2, 25.9, 24.0, 23.5. LCMS (*m/z*): $[M+H]^+$ calc'd for $C_{37}H_{39}ClN_6O_5$ 683.27; found, 683.45

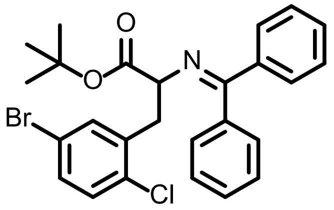

***tert*-butyl 3-bromo-6-chloro-*N*-(diphenylmethylidene)phenylalaninate**: To a solution of *tert*-butyl *N*-(diphenylmethylidene)glycinate (6.75 g, 22.9 mmol) and *N*-benzyl-*N*,*N*-diethylethanaminium chloride (1.56 mg, 6.86 mmol) in dichloromethane (100 mL) was added sodium hydroxide (4.57 g, 114 mmol) at 0 °C. The reaction was allowed to stir for 10 minutes before the addition of a solution of 4-bromo-2-(bromomethyl)-1-chlorobenzene (6.50 mg, 22.86 mmol) in dichloromethane (5 ml). The reaction was stirred at 15 °C for 16 hours. The reaction mixture was then diluted with dichloromethane (100 mL) and washed with water (200 mL). The organic phase was concentrated and the residue was purified via flash column (silica gel, 40 g, EA/PE=0~5 %) to provide pure title compound (6.4 g) as a yellow solid. ^{1}H NMR (400MHz, $CDCl_3$-*d*) δ ppm 7.57 (s, 2H), 7.41 - 7.28 (m, 7H), 7.26 - 7.20 (m, 1H), 7.10 (d, *J*=8.5 Hz, 1H),

6.83 - 6.69 (m, 2H), 4.32 - 4.24 (m, 1H), 3.35 (d, *J*=4.5 Hz, 1H), 3.28 - 3.17 (m, 1H), 1.46 (s, 9H). LCMS (*m/z*): $[M+H]^+$ calc'd for $C_{26}H_{25}BrClNO_2$, 498.853; found, 500.23.

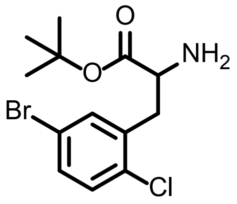

***tert*-butyl 3-bromo-6-chlorophenylalaninate**: To a solution of *tert*-butyl 3-bromo-6-chloro-*N*-(diphenylmethylidene)phenylalaninate (2000 mg, 4.01 mmol) in tetrahydrofuran (20 mL) was added a 0.5 N citric acid solution (32 mL, 16 mmol) and the reaction was stirred at room temperature for 18 hours. The reaction was then basified with sodium bicarbonate and extracted with dichloromethane. The organic phase was dried over anhydrous Na_2SO_4, filtered and concentrated *in vacuo* to afford the crude product which was purified by column chromatography (silica gel, 40 g, ethyl acetate/heptane 0-80%) to provide the title compound (1095 mg, 82%) as a colorless oil. ^{1}H NMR (500 MHz, DMSO-d_6) δ ppm 7.56 (d, *J*=2.45 Hz, 1 H) 7.46 (dd, *J*=8.31, 2.45 Hz, 1 H) 7.39 (d, *J*=8.56 Hz, 1 H) 3.49 (t, *J*=7.46 Hz, 1 H) 2.94 (dd, *J*=13.45, 7.09 Hz, 1 H) 2.83 (dd, *J*=13.57, 7.70 Hz, 1 H) 1.83 (s, 2 H) 1.33 (s, 9 H). ^{13}C NMR (126 MHz, DMSO-d_6) δ ppm 174.56, 139.10, 134.97, 133.07, 131.40, 131.33, 120.09, 80.50, 54.95, 38.87, 28.02. HRMS (*m/z*): $[M+H]^+$ calc'd for $C_{13}H_{18}BrClNO_2$, 334.0203; found, 334.0204.

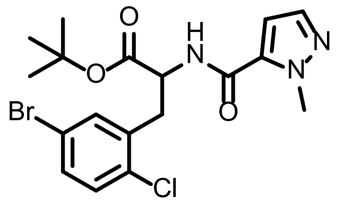

***tert*-butyl-3-bromo-6-chloro-*N*-[(1-methyl-1*H*-pyrazol-5-yl)carbonyl]phenylalaninate**: To a solution of *tert*-butyl 3-bromo-6-chlorophenylalaninate (1080 mg, 3.23 mmol), 1-methyl-1*H*-pyrazole-5-carboxylic acid (448 mg, 3.55 mmol) and DIPEA (2.82 mL, 16.1 mmol) in DMF (15 mL) was added HATU (1520 mg, 3.87 mmol). The reaction was allowed to stir for 3 hours at room temperature. The reaction was extracted with ethyl acetate and water. The organics were separated, dried over sodium sulfate, filtered and the solvent removed. The crude product was purified by column chromatography (silica gel, 40 g, ethyl acetate/heptane 0-60%) to provide **C**

(1360 mg, 95%) as a yellow solid. ^{1}H NMR (500 MHz, DMSO-d_6) δ ppm 8.81 (d, *J*=8.07 Hz, 1 H) 7.63 (d, *J*=2.45 Hz, 1 H) 7.45 - 7.50 (m, 2 H) 7.39 - 7.43 (m, 1 H) 6.88 (d, *J*=1.96 Hz, 1 H) 4.63 (ddd, *J*=9.60, 8.13, 5.99 Hz, 1 H) 3.28 - 3.35 (m, 2 H) 3.04 - 3.13 (m, 1 H) 1.38 (s, 9 H). ^{13}C NMR (126 MHz, DMSO-d_6) δ ppm 170.3, 159.9, 138.0, 137.7, 135.1, 134.9, 133.1, 131.8, 131.6, 120.2, 108.0, 81.6, 52.5, 39.2, 34.6, 28.0. LCMS (*m/z*): $[M+H]^+$ cal'd. for $C_{18}H_{21}BrClN_3O_3$, 441.045; found, 442.19.

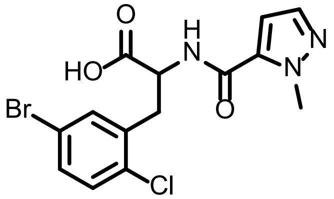

3-bromo-6-chloro-*N*-[(1-methyl-1*H*-pyrazol-5-yl)carbonyl]phenylalanine:

To a solution of *tert*-butyl 3-bromo-6-chloro-*N*-[(1-methyl-1*H*-pyrazol-5-yl)carbonyl]phenylalaninate (1.07 g, 2.40 mmol) in DCM (20 mL) was added TFA (20 mL, 260 mmol). The reaction was stirred for 3 hours at room temperature. The solvent was then removed *in vacuo* and the residue was taken up in sodium hydrogen sulfate and extracted with ethyl acetate. The organic layer was dried over anhydrous Na_2SO_4, filtered and the solvent was removed *in vacuo* to afford the title compound (927 mg, 99%) as an off-white solid. ^{1}H NMR (500 MHz, DMSO-*d*6) δ ppm 8.77 (d, J=8.8 Hz, 1H), 7.63 (d, J=2.4 Hz, 1H), 7.50 - 7.43 (m, 2H), 7.42 - 7.38 (m, 1H), 6.85 (d, J=2.2 Hz, 1H), 4.71 (ddd, J=4.4, 8.6, 11.0 Hz, 1H), 3.94 (s, 3H), 3.39 (dd, J=4.4, 13.7 Hz, 1H), 3.07 (dd, J=11.0, 13.9 Hz, 1H). ^{13}C NMR (126 MHz, DMSO-d_6) δ ppm 172.8, 159.8, 138.3, 137.7, 135.1, 134.9, 133.1, 131.7, 131.6, 120.1, 107.9, 51.5, 39.2, 34.6. HRMS (*m/z*): $[M+H]^+$ calc'd for $C_{14}H_{14}BrClN_3O_3$, 385.9901; found, 385.9902.

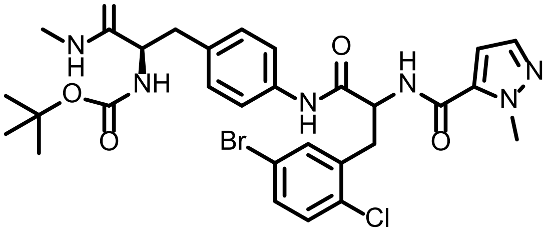

***tert*-butyl[(2*R*)-3-(4-{[3-(5-bromo-2-chlorophenyl)-2-{[(1-methyl-1*H*-pyrazol-5-yl)carbonyl]amino}propanoyl]amino}phenyl)-1-(methylamino)-1-oxopropan-2-**

yl]carbamate: To a solution of 3-bromo-6-chloro-*N*-[(1-methyl-1*H*-pyrazol-5-yl)carbonyl]phenylalanine (670 mg, 1.73 mmol), 4-amino-*N*a-(*tert*-butoxycarbonyl)-*N*-methyl-D-phenylalaninamide (610 mg, 2.08 mmol) and DMAP (212 mg, 1.73 mmol) in DCM (17 mL) was added EDCI (400 mg, 2.08 mmol). The reaction was stirred for 18 hours at room temperature. The reaction was then extracted with ethyl acetate and water. The organics were dried over anhydrous Na_2SO_4, filtered and the solvent removed *in vacuo*. The crude product was purified by column chromatography (silica gel, 120 g, methanol/ethyl acetate 0-5%) to provide the title compound (687 mg, 60%) as an off-white solid. ^{1}H NMR (500 MHz, DMSO) δ (ppm) δ = 10.05 (s, 1H), 8.78 (d, *J*=8.6 Hz, 1H), 7.81 (br. s., 1H), 7.66 (s, 1H), 7.53 - 7.37 (m, 5H), 7.17 (d, *J*=8.1 Hz, 2H), 6.95 (s, 1H), 6.85 (d, *J*=8.3 Hz, 1H), 4.98 - 4.88 (m, 1H), 4.12 - 4.06 (m, 1H), 3.97 (s, 3H), 3.30 (d, *J*=5.1 Hz, 1H), 3.19 - 3.12 (m, 1H), 2.94 - 2.85 (m, 1H), 2.73 - 2.66 (m, 1H), 2.59 (br. s., 3H), 1.32 (s, 9H). ^{13}C NMR (126 MHz, DMSO-d_6): δ (ppm) 172.4, 169.4, 159.9, 155.7, 138.2, 137.6, 135.2, 134.5, 133.3, 131.6, 131.5, 129.8, 120.1, 108.2, 78.4, 53.1, 39.3, 38.7, 28.7, 26.1. LCMS (*m/z*): $[M+H]^+$ calc'd for $C_{29}H_{34}BrClN_6O_5$, 661.99; found, 663.30.

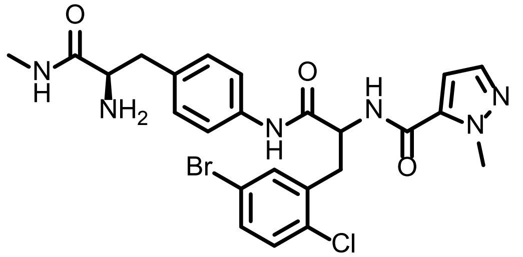

***N*-[1-({4-[(2*R*)-2-amino-3-(methylamino)-3-oxopropyl]phenyl}amino)-3-(5-bromo-2-chlorophenyl)-1-oxopropan-2-yl]-1-methyl-1*H*-pyrazole-5-carboxamide**: To a solution of [(2*R*)-3-(4-{[3-(5-bromo-2-chlorophenyl)-2-{[(1-methyl-1*H*-pyrazol-5-yl)carbonyl]amino}propanoyl]amino}phenyl)-1-(methylamino)-1-oxopropan-2-yl]carbamate in dichloromethane (10 mL) was added a 4 N solution of HCl in dioxane (10 mL). The reaction was stirred for 2 hours at room temperature. The solvent was then removed *in vacuo* and the resulting residue was taken up a dilute sodium bicarbonate solution and extracted with ethyl acetate. The organic layer was dried over anhydrous Na_2SO_4, filtered and conc. *in vacuo* to provide the title compound (169 mg, 92%) as a yellow solid. ^{1}H NMR (500 MHz, DMSO-d_6) δ ppm 10.15 (s, 1 H) 8.81 (d, *J*=8.31 Hz, 1 H) 8.27 (d, *J*=4.40 Hz, 1 H) 8.09 - 8.20 (m, 3 H) 7.66 (d, *J*=1.96 Hz, 1 H) 7.57 (d, *J*=8.31 Hz, 2 H) 7.43 - 7.50 (m, 2 H) 7.38 - 7.43 (m, 1 H) 7.15 (d,

J=8.56 Hz, 2 H) 6.95 (d, *J*=1.96 Hz, 1 H) 4.87 - 4.96 (m, 1 H) 3.97 (s, 3 H) 3.87 (dd, *J*=11.49, 7.09 Hz, 1 H) 3.32 (dd, *J*=14.18, 5.38 Hz, 1 H) 3.16 (dd, *J*=13.94, 9.54 Hz, 1 H) 2.88 - 3.03 (m, 2 H) 2.70 (s, 1 H) 2.61 (d, *J*=4.65 Hz, 3 H). ^{13}C NMR (126 MHz, DMSO-d_6) δ ppm 169.65, 168.64, 159.93, 138.13, 137.65, 135.18, 134.45, 133.32, 131.68, 131.53, 130.56, 130.10, 120.44, 120.16, 108.27, 54.20, 53.28, 39.26, 38.73, 36.99, 35.16, 26.00. HRMS (*m/z*): $[MH]^+$ calc'd for $C_{24}H_{27}BrClN_6O_3$, 561.1011; found, 561.1001.

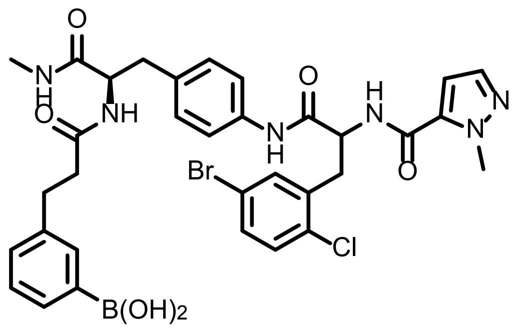

[3-(3-{[(2*R*)-3-(4-{[3-(5-bromo-2-chlorophenyl)-2-{[(1-methyl-1*H*-pyrazol-5-yl)carbonyl]amino}propanoyl]amino}phenyl)-1-(methylamino)-1-oxopropan-2-yl]amino}-3-oxopropyl)phenyl]boronic acid: To a solution of *N*-[1-({4-[(2*R*)-2-amino-3-(methylamino)-3-oxopropyl]phenyl}amino)-3-(5-bromo-2-chlorophenyl)-1-oxopropan-2-yl]-1-methyl-1*H*-pyrazole-5-carboxamide (92 mg, 0.16 mmol), 3-(3-boronophenyl)propionic acid (38 mg, 0.196 mmol) and DIPEA (0.14 mL, 0.819 mmol) in DMF (2 mL) was added HATU (77 mg, 0.196 mmol). The reaction was stirred for 1 hour at room temperature. The solvent was removed *in vacuo* and the crude product was purified by column chromatography (silica gel, 12 g, methanol/dichlormethane 0-15%) to provide the title compound (78 mg, 65%) as a white solid. ^{1}H NMR (500 MHz, DMSO-d_6) δ ppm 10.07 (s, 1 H) 8.78 (d, *J*=8.56 Hz, 1 H) 8.28 (br. s., 2 H) 8.09 (d, *J*=8.31 Hz, 1 H) 7.95 - 8.02 (m, 2 H) 7.82 (dd, *J*=4.52, 2.32 Hz, 1 H) 7.57 - 7.67 (m, 3 H) 7.46 - 7.51 (m, 2 H) 7.43 (dd, *J*=8.56, 2.45 Hz, 1 H) 7.38 (d, *J*=8.31 Hz, 1 H) 7.19 - 7.27 (m, 2 H) 7.13 - 7.18 (m, 2 H) 6.94 (d, *J*=1.96 Hz, 1 H) 4.92 (td, *J*=9.05, 5.38 Hz, 1 H) 4.42 (td, *J*=8.86, 5.50 Hz, 1 H) 3.96 (s, 3 H) 3.11 - 3.19 (m, 4 H) 2.93 (d, *J*=5.14 Hz, 1 H) 2.79 - 2.88 (m, 1 H) 2.56 (d, *J*=4.65 Hz, 3 H) 2.33 - 2.42 (m, 2 H). ^{13}C NMR (126 MHz, DMSO-d_6): δ = 174.3, 172.0, 171.7, 169.5, 162.8, 159.9, 140.5, 140.1, 138.2, 137.6, 137.2, 135.2, 134.5, 133.3, 132.1, 131.5, 130.4, 130.3, 129.7, 127.8, 127.8, 120.1, 108.2, 54.1, 42.3, 39.3, 38.7, 26.0, 18.6, 17.2, 13.0 ppm. HRMS (*m/z*): $[MH]^+$ calc'd for $C_{33}H_{36}BBrClN_6O_6$, 737.1662; found, 737.1642.

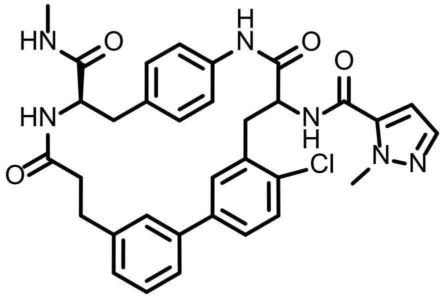

Compound 3: (9R)-24-chloro-N-methyl-4-(1-methyl-1H-pyrazole-5-carboxamido)-5,11-dioxo-6,10-diaza-1,2(1,3),7(1,4)-tribenzenacyclotridecaphane-9-carboxamide: To a solution of [3-(3-{[(2*R*)-3-(4-{[3-(5-bromo-2-chlorophenyl)-2-{[(1-methyl-1*H*-pyrazol-5-yl)carbonyl]amino}propanoyl]amino}phenyl)-1-(methylamino)-1-oxopropan-2-yl]amino}-3-oxopropyl)phenyl]boronic acid (75 mg, 0.10 mmol) and potassium fluoride (29.5 mg, 0.508 mmol) in THF:water (0.6 mL:0.2 mL) was added Pd(dppf)Cl_2 (3.8 mg, 0.00508 mmol). The reaction was stirred for 3 hours at 70 °C. The reaction was then filtered and the solvent was removed *in vacuo*. The crude product was purified by column chromatography (C18, 12 g, acetonitrile/water with 0.1% formic acid 5-50%) to provide the title compound (1.9 mg, 3%) as a white solid. ^{1}H NMR (600 MHz, DMSO-d_6) δ 9.93 (s, 1H), 8.87 (d, *J* = 6.9 Hz, 1H), 8.02 (d, *J* = 8.9 Hz, 1H), 7.88 (q, *J* = 4.6 Hz, 1H), 7.52 (m, 1H), 7.50 (d, *J* = 2.1 Hz, 1H), 7.43 (dd, *J* = 8.3, 2.3 Hz, 1H), 7.33 – 7.29 (m, 4H), 7.26 (d, *J* = 2.3 Hz, 1H), 7.23 (d, *J* = 7.2 Hz, 1H), 7.10 (d, *J* = 2.1 Hz, 1H), 7.06 (d, *J* = 8.2 Hz, 2H), 6.64 (s, 1H), 4.73 (m, 1H), 4.19 (ddd, *J* = 12.1, 8.9, 3.0 Hz, 1H), 4.06 (s, 3H), 3.34 - 3.20 (m, 2H), 2.94 (m, 1H), 2.84 (m, 1H), 2.60 (d, *J* = 4.6 Hz, 3H), 2.55 (m, 2H), 2.47 (m, 1H), 2.38 (m, 1H). ^{13}C NMR (150 MHz, DMSO-d_6) δ 172.0, 171.4, 169.2, 159.1, 139.5, 138.8, 137.1, 136.5, 134.8, 134.7, 133.9, 133.6, 132.6, 129.8, 129.7, 129.2, 129.2, 128.9, 127.4, 127.0, 126.8, 124.3, 118.9, 118.9, 107.7, 53.7, 53.6, 38.7, 36.6, 36.4, 35.5, 31.7, 25.4. HRMS (*m/z*): $[MH]^+$ calc’d for $C_{33}H_{33}ClN_6O_4$, 613.2325; found, 613.2342.

Supplementary Method

Expression and purification of ^{15}N, ^{13}C enriched IL-17A protein:

IL-17A protein used in the NMR studies was expressed in *E.coli* BL21 cells that were transfected with pXC227 vector encoding IL17A gene. The bacteria were grown in minimal media containing $^{15}NH_4Cl$ and $^{13}C_6$-glucose as sole nitrogen and carbon sources to make uniformly ^{13}C and ^{15}N-enriched protein.

Inclusion body protein was solubilized by homogenization into a solubilization buffer [50 mM Tris (pH 8.5), 6 M guanidine HCl, and 10 mM DTT] at room temperature for 1 h. Solubilized protein was refolded at a final concentration of 0.5 mg/ml by rapid dilution into a refold buffer with vigorous stirring [0.1 M CAPSO (pH 9.5), 0.9 M arginine, and 0.3:0.03 mM reduced/oxidized glutathione] at room temperature and left to stand overnight. Refolded protein was concentrated 5-fold using a 10 kDa molecular weight cutoff spiral cartridge (Amicon proflux™ M12 Tangential flow system). (Protein loss was incurred in the past hence the protein was filtered and spun down and concentrated on VIVA SPIN 10k DA MW cut off membrane.

Concentrated protein was purified by size-exclusion chromatography using S75 26/60 16/60 (GE Healthcare Biosciences) columns equilibrated and developed with 50 mM Tris (pH 7.4) and 0.15 M NaCl.

**Compounds used**[*]	Compound **1**	Compound **2**	Compound **3**
PDB code	5HI3	5H4	5HI5
Space group	$P2_1$	$P2_1$	$P2_1$
Unit cell (Å, °)	91.11 68.32 100.45<br>90.00 90.82 90.00	91.24 68.19 100.12<br>90.00 91.33 90.00	91.32 68.68 100.53<br>90.00 90.65 90.00
Resolution (Å)	47.8-2.15(2.28-2.15)	50.05-1.8(1.97-1.8)	91.31-1.8(2.08-1.8)
# of unique reflections	63,945	112,879	113,034
I/σ	16.7(3.5)	169.6(17.8)	19.9(4.6)
Rmerge	0.053/0.344	0.056(0.750)	0.037(0.408)
Completeness (%)	94.6(74.0)	99.1(99.6)	97.9(97.5)
multiplicity	3.0(2.1)	2.8(2.7)	3.4(3.4)
Rwork/Rfree	0.197/0.231	0.199/0.224	0.201/0.219
Deviation from ideal geometry,<br>Bond length (Å)<br>/angle (º)	0.01, 1.14	0.010,1.12	0.010,1.08

Table S1. X-ray crystallographic data collection and refinement statistics

[*] Numbers in parenthesis are for highest resolution shell.

Supplementary Figures

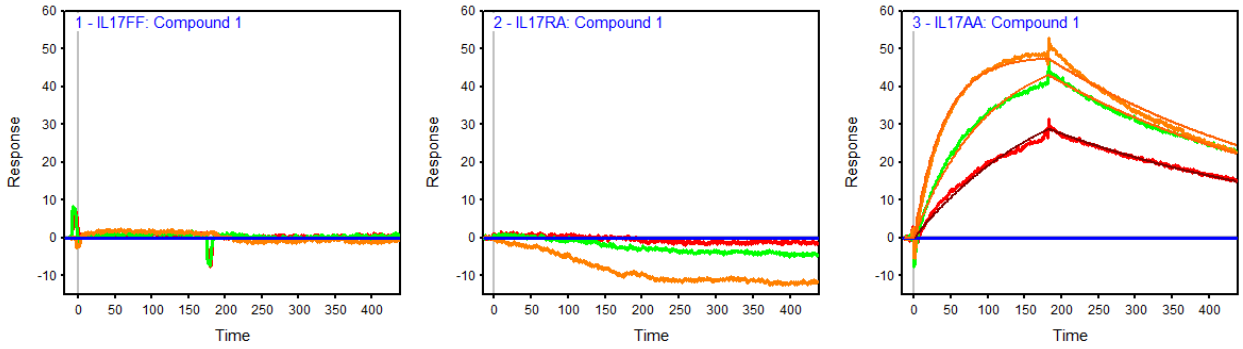

Supplementary Figure S1. SPR binding response of Compound 1 to IL-17F, IL-17RA and IL-17A. The highest concentration tested was at top concentration of 40 μM along with a 3-fold dilution series. Compound **1** clearly shows binding to IL-17A with no measurable binding to IL-17F and IL-17RA at these concentrations.

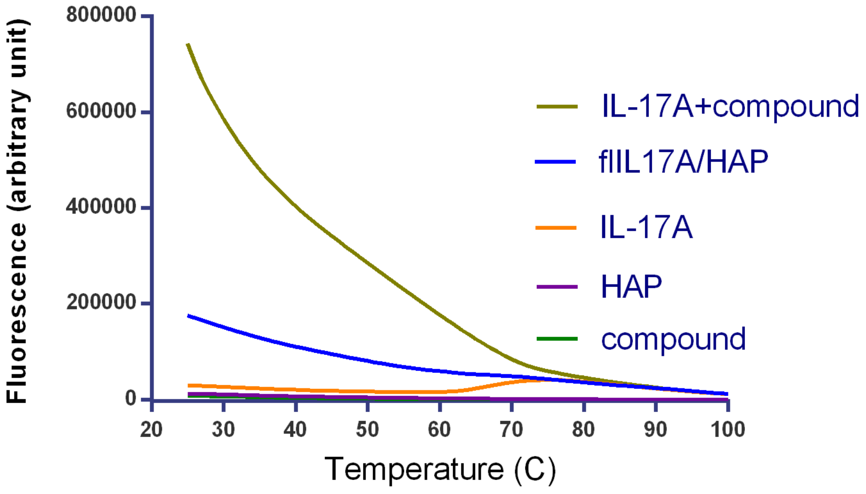

S2. A

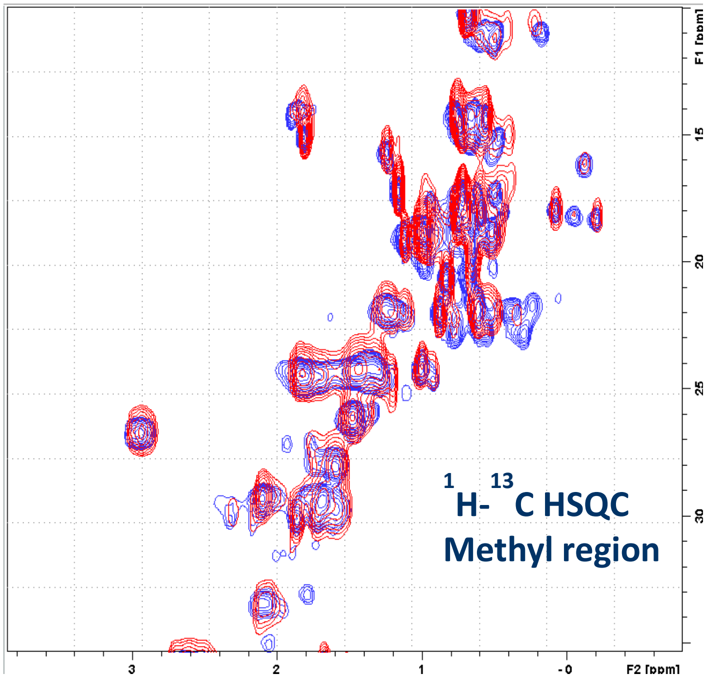

S2. B

Figure S2. IL-17A becomes more flexible in the presence of compounds. A. Typical thermal melting curves of IL-17A in presence of representative compound or HAP. Without compound or HAP, full length IL-17A behaves as a well folded protein with a Tm of 67 °C. In the presence of a compound, IL-

17A becomes much more loosely packed and has strong fluorescent absorption even at room temperature. In the presence of HAP, IL-17A also becomes less packed but to a much lesser degree than observed with compound. HAP and compound themselves did not have significant fluorescence. B. Overlay of methyl region from ^{1}H -^{13}C NMR HSQC correlation spectra for uniformly ^{15}N, ^{13}C labeled apo IL-17A in blue and IL-17A + representative compound in red. In the presence of compound, methyl signals of IL-17A show extensive line broadening indicating conformational exchange and enhanced flexibility on an intermediate timescale in NMR.

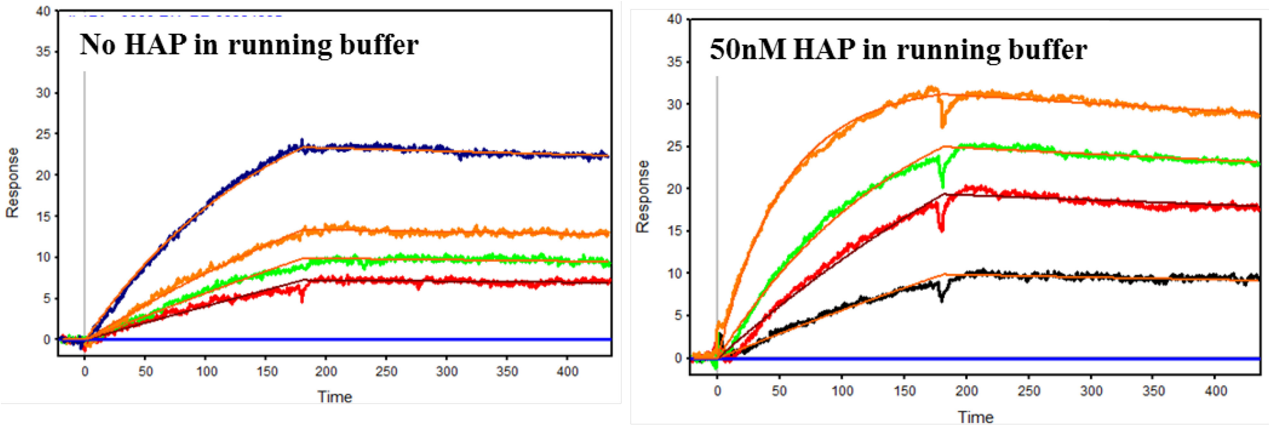

K_D = 41.2 nM
k_{on} = 4.169E+03 $M^{-1}s^{-1}$
k_{off} = 1.724E-04 s^{-1}

K_D = 23.8 nM
k_{on} = 1.253E+04 $M^{-1}s^{-1}$
k_{off} = 2.980E-04 s^{-1}

Figure S3: SPR sensorgrams shown for 4 concentrations of a representative linear compound binding to immobilized IL-17A in absence (left) and presence (right) of HAP in running buffer. In absence of HAP, compound binds to IL-17A with an approximately 3 fold slower on-rate relative to IL-17A with a saturating concentration of HAP in the running buffer.

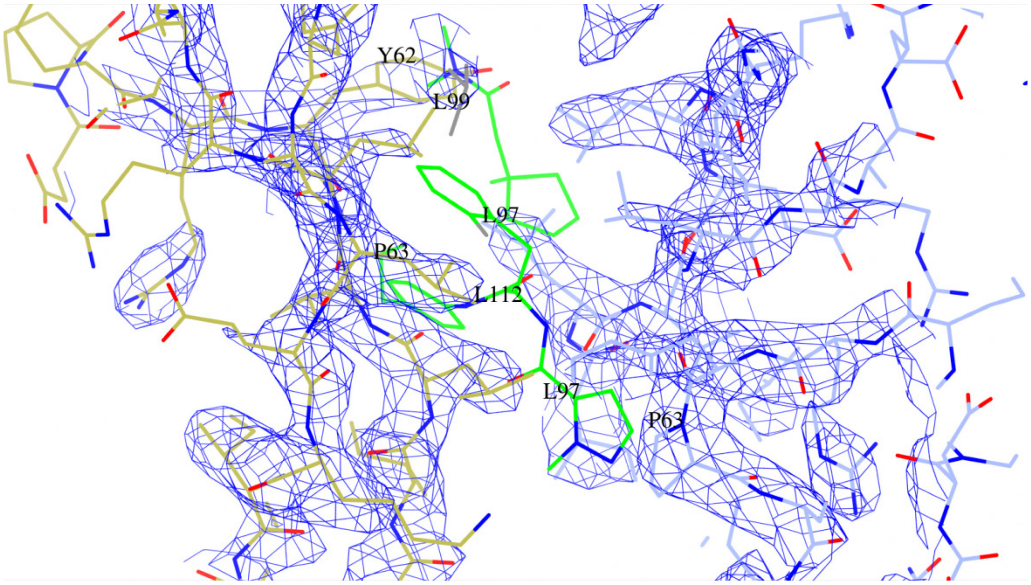

S4 A.

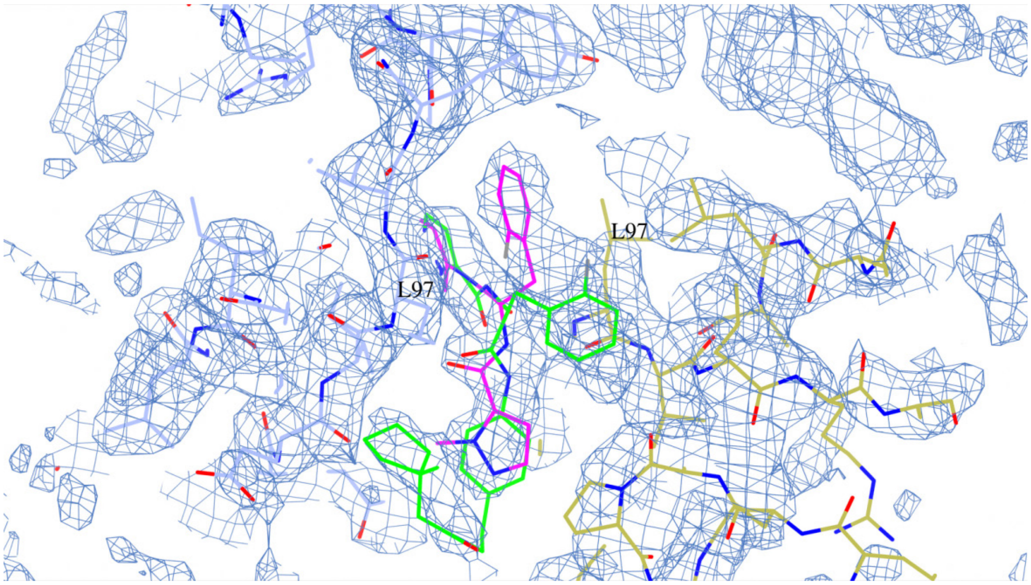

S4B.

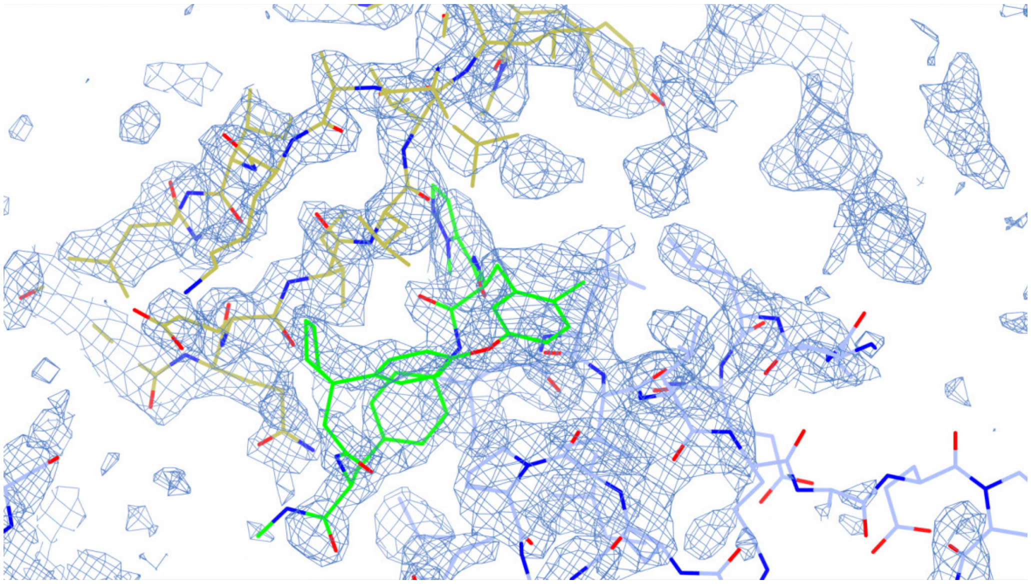

S4C.

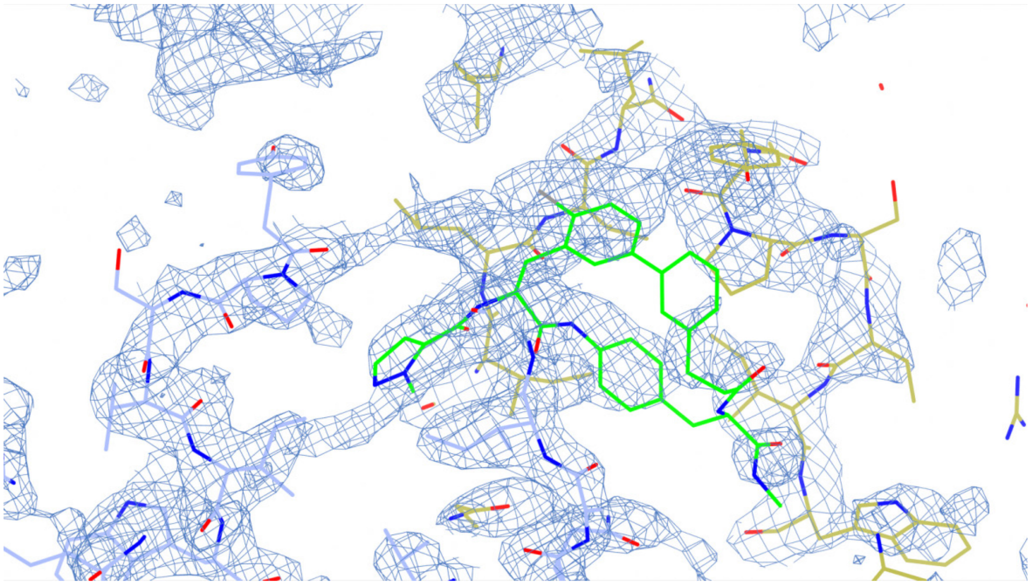

S4D

Figure S4. Electron densities at the IL-17A compound binding site. A. A closed, unoccupied compound binding site when 1 mM compound **1** was co-crystallized with Fab/IL-17A binary complex. IL-17A (bonds represented in lines, with carbon atoms colored light blue and gold for different monomer chain, and nitrogen colored in blue, oxygen in red) is embedded in well-defined electron density (blue mesh, contoured at 1.0 σ). There is no density for compound **1**

(carbon atoms in green) found at the open binding site, and the compound clashes with protein atoms. B. Compound **1** binds at an enlarged central pocket in IL-17A in the Fab/HAP/IL-17A complex. Because IL-17A dimer is symmetric, compound **1** binds with two poses with near equal occupancies (magenta and green carbons). These two poses have a pseudo 2-fold symmetry with the Cα atom near the axis. C. compound **2** binds at the same opened binding pocket of IL-17A in the Fab/HAP/IL-17A complex. D. Compound **3** binds at the same opened binding pocket of IL-17A in the Fab/HAP/IL-17A complex. Notice that the cyclization linker of compound **3** has weaker electron density probably due to flexibility and loose fitting at the binding site. Probably due to subtle difference in crystal packing environment, compound **2** and **3** bind to IL-17A dimer with one dominant pose.

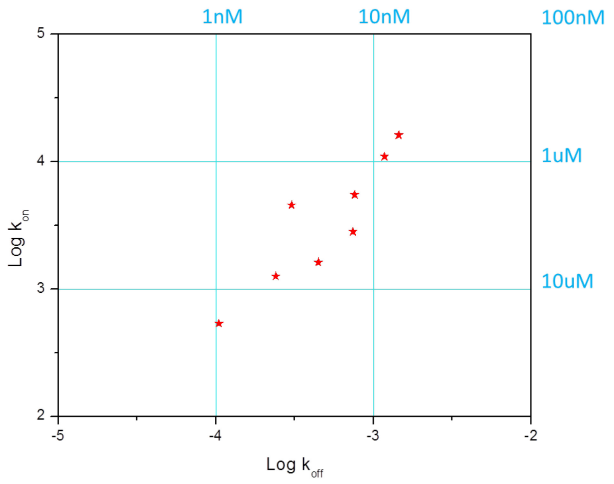

Figure S5: 3 dimensional plot showing correlation of off-rates (x-axis), on-rates (y-axis) and K_D (diagonal axis) for 7 compounds in the linear compound series (red stars). Slow on-rates are observed with a compensatory decrease in off-rates, resulting in similar K_D ($K_D = k_{off}/k_{on}$).

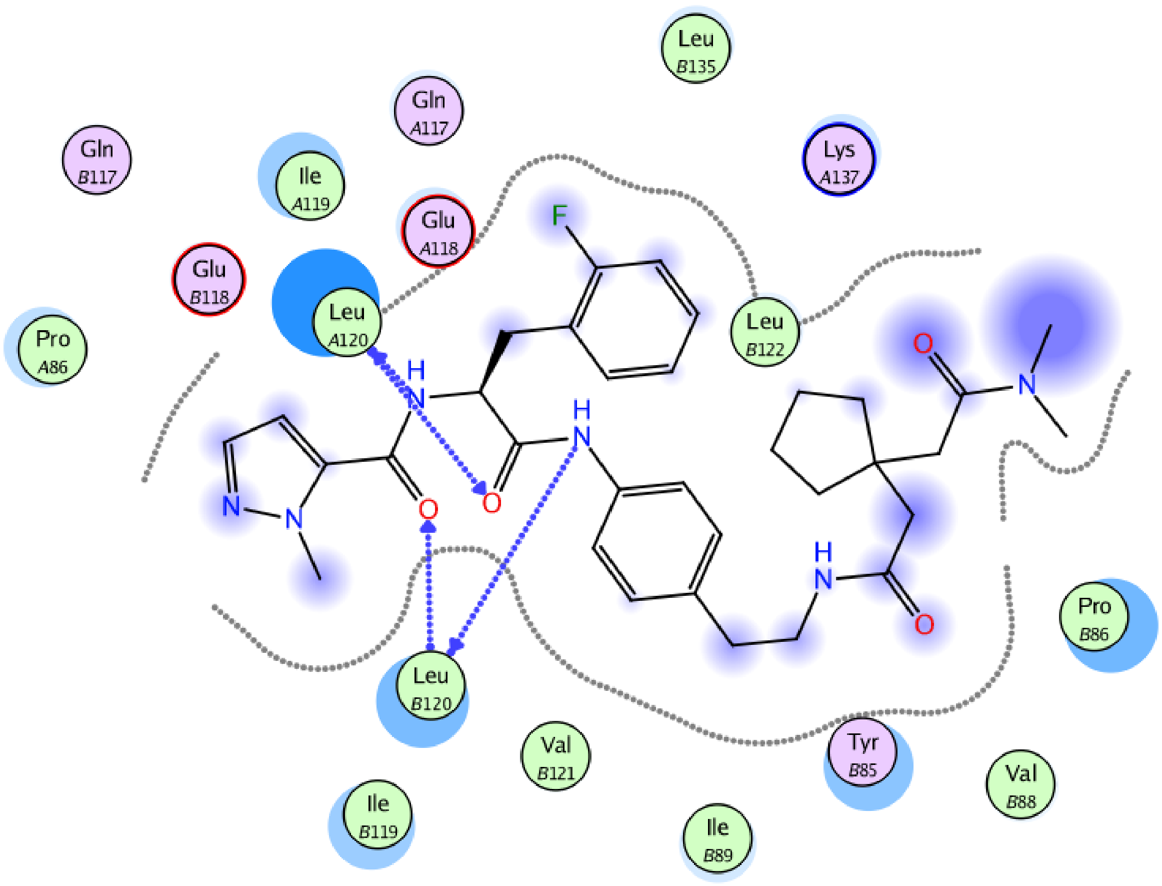
Leu B135
Gln A117
Lys A137
Gln B117
Ile A119
Glu A118
Glu B118
Leu A120
Pro A86
Leu B122
F
H N
O
N
O
N
H N
O
H N
O
N N
Leu B120
Pro B86
Val B121
Tyr B85
Val B88
Ile B119
Ile B89

S6A

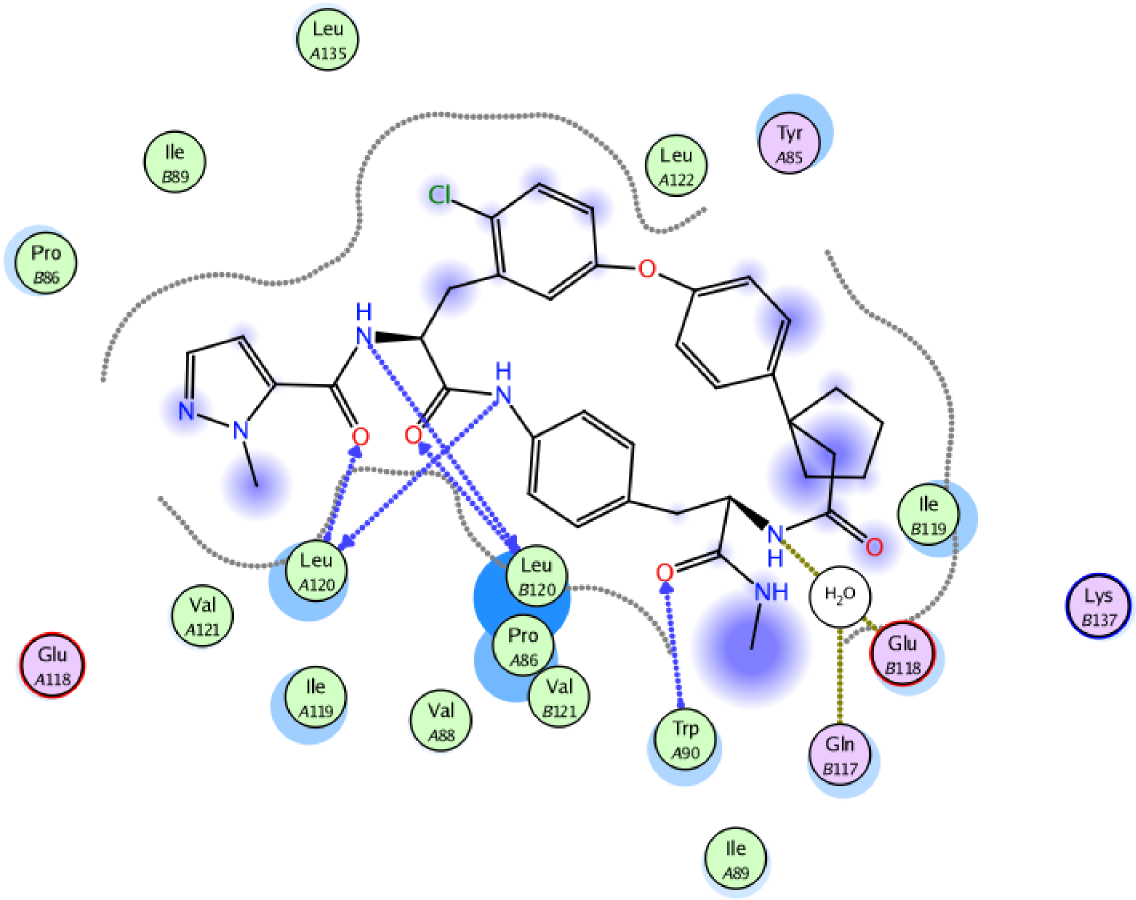
Leu A135
Ile B89
Pro B86
Leu A122
Tyr A85
Cl
O
H N
H N
N
N
O
O
Ile B119
N H
O
H2O
O
NH
Lys B137
Leu A120
Leu B120
Val A121
Glu A118
Pro A86
Val B121
Glu B118
Ile A119
Val A88
Trp A90
Gln B117
Ile A89

S6B

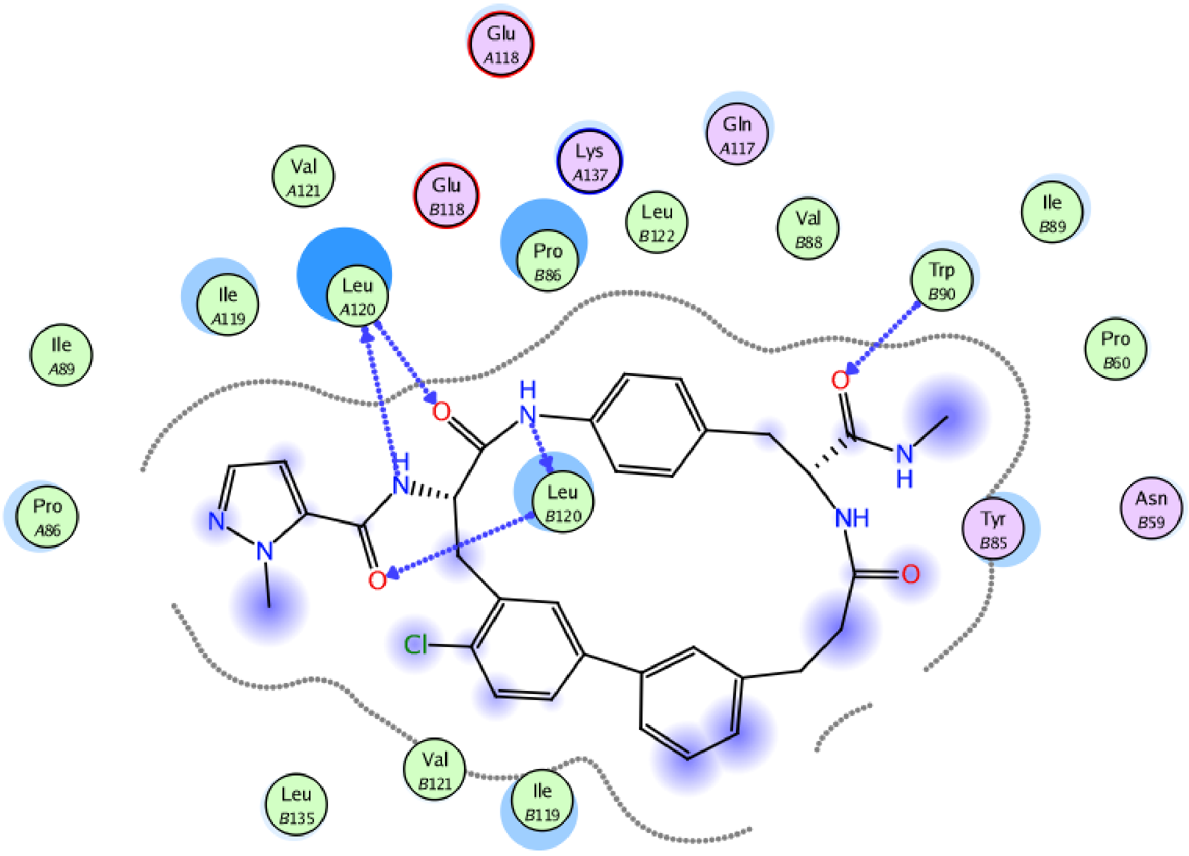

S6C

Figure S6. Schematic presentations of compound **1** (S6A), **2** (S6B) and **3** (S6C) interacting with IL-17A binding site. Hydrogen bonds are shown in blue dashed arrow pointing from donor to acceptor. Binding site proximity contour are shown in black dashes. Sizes of the blue shadows near protein residue symbols and haloes around ligand atoms are proportional to degree of their solvent exposures.

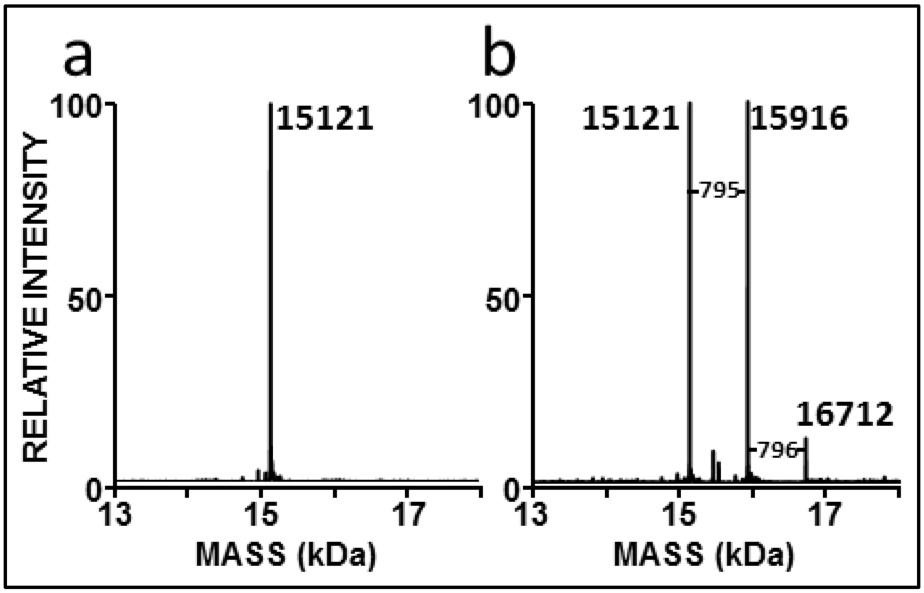

Figure S7. Mass spectra of IL-17A monomers from protein (a) before and (b) after treatment with compound **4**. The interchain disulfide bonds were reduced with DTT following exposure of IL-17A to the sulfonyl fluoride probe. The mass shift predicted to result from a single modification of the polypeptide was 795.3 Da.

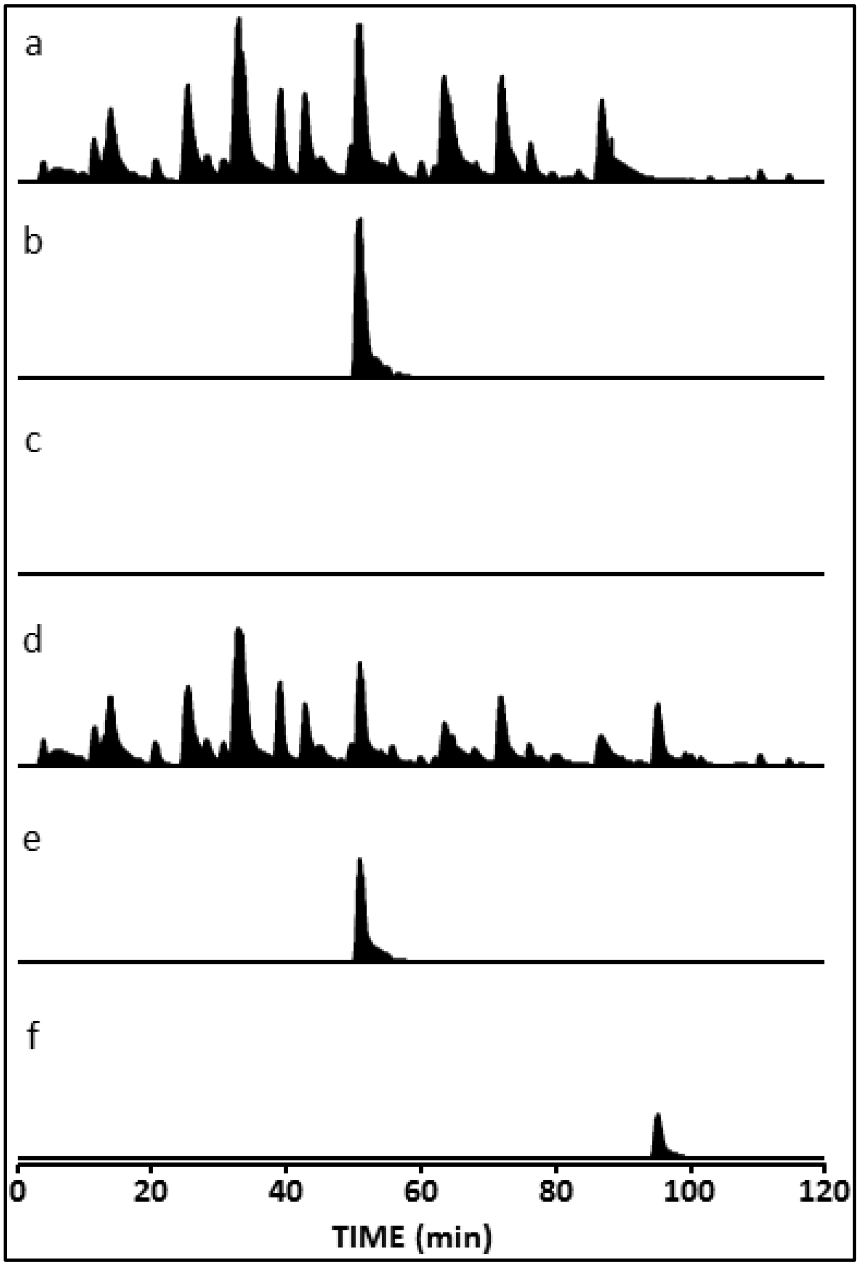

Figure S8. LC-MS peptide mapping analysis of IL-17A before and after treatment with compound **4**. All plots are on the same scale of intensity (normalized level 2.8E7). Panels a-c show data from a Lys-C/trypsin digest of untreated protein. (a) Base peak chromatogram; (b) extracted ion plot for peptide 79-93 of IL-17A (sequence NEDPERYPSVIWEAK: $[M+2H]^{2+}$ theor.= 916.94, extracted ion plot given for *m/z* = 916.56-917.36); (c) extracted ion plot showing no signal for peptide 79-93 modified by compound **4** ($[M+2H]^{2+}$ theor.= 1315.1, extracted ion plot given for 1314.1-1315.1). Panels d-f show data from a Lys-C/tryptic digest of treated protein. (d) Base peak chromatogram; (e) extracted ion plot for peptide 79-93 of IL-17A as in panel b; (f) extracted ion plot for peptide 79-93 modified by compound **4** as in panel c. The peptide mapping data agree well with modification of one polypeptide in each IL-17A homodimer.

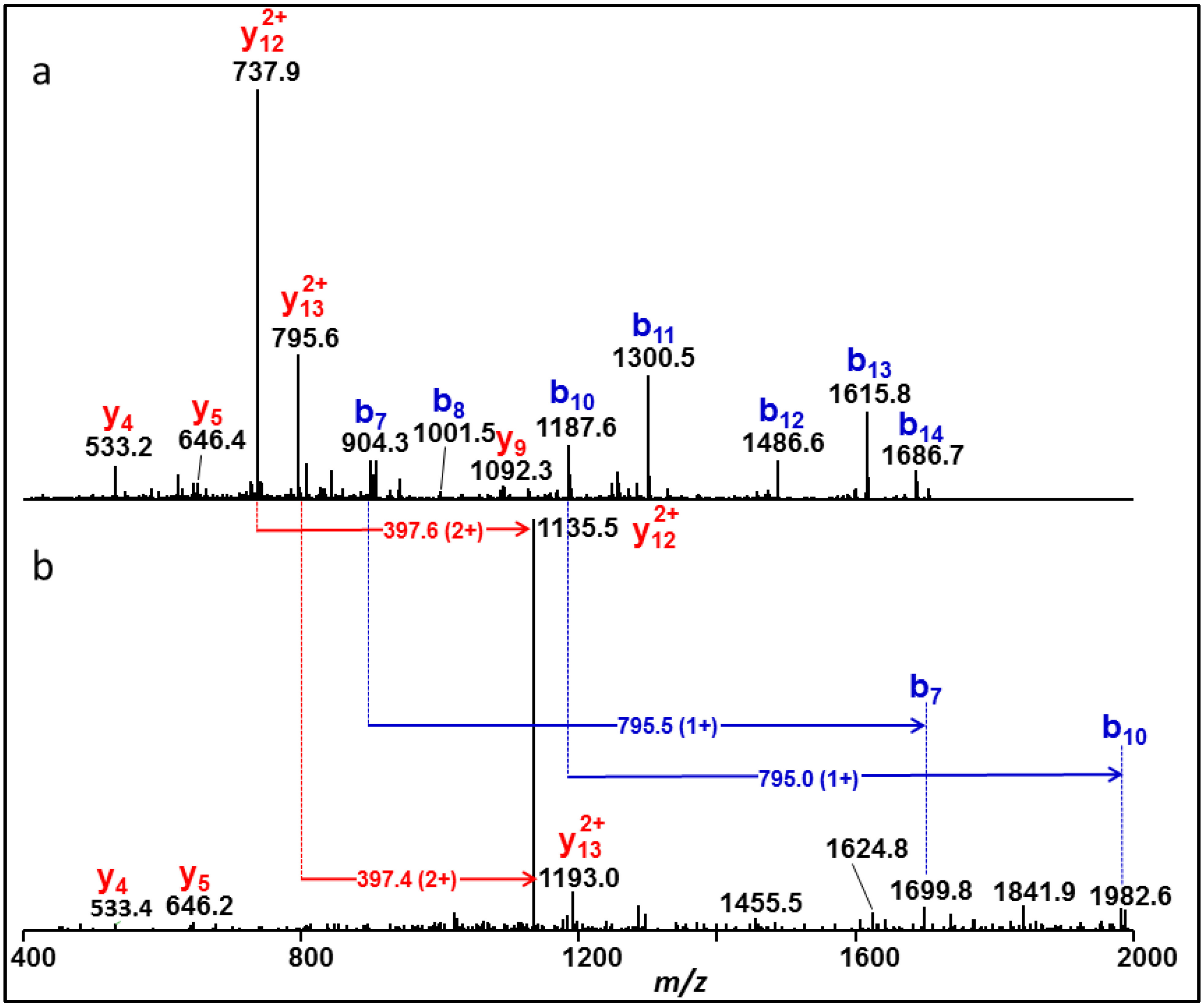

Figure S9. Tandem MS spectra showing that compound **2** modified IL-17A at Tyr-85. (a) MS/MS spectrum interpreted as originating from Lys-C/tryptic peptide 79-93 of IL-17A (sequence NEDPERYPSVIWEAK). (b) MS/MS spectrum from analysis of modified IL-17A interpreted as originating from peptide 79-93 modified at Tyr-85. Mass shifts in significant ions are indicated, and agree with the theoretical mass shift caused by modification with compound **2** of 795.3 Da.

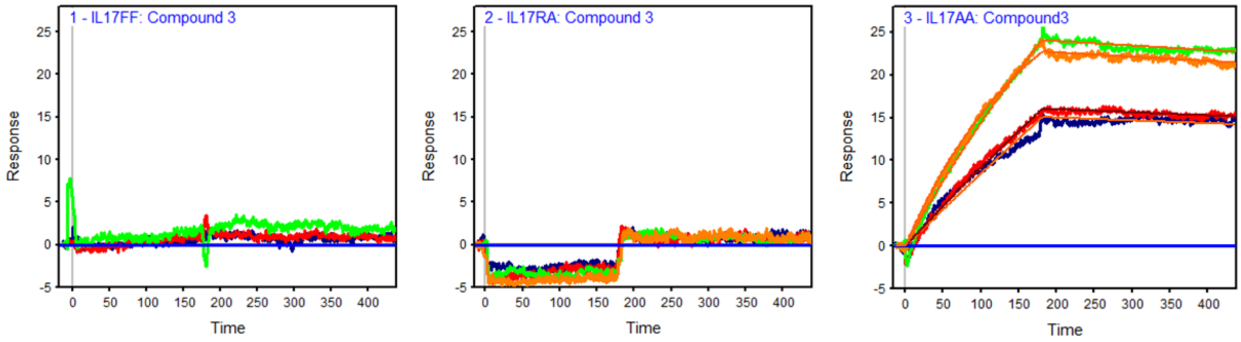

Supplementary Figure S10. SPR binding response of Compound **3** to IL-17F, IL-17RA and IL-17A. The highest concentration tested was 13.3 μM along with a 3-fold dilution series. Compound **3** clearly shows binding to IL-17A while no measurable binding to IL-17F and IL-17RA at these concentrations. Compound **2** has similar SPR profiles as compound **3**.

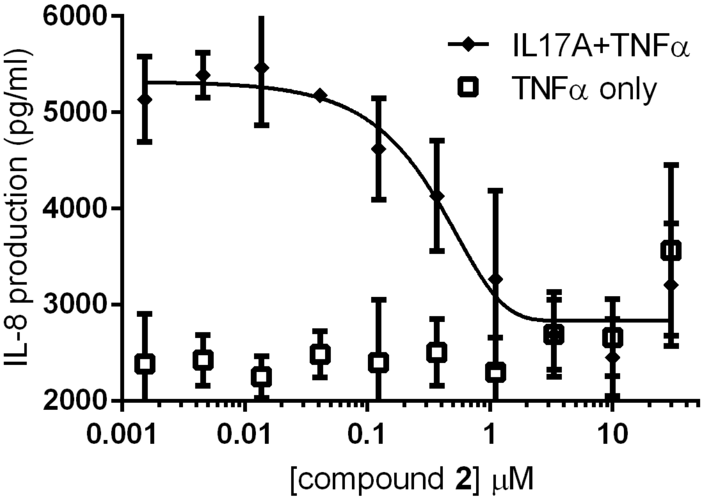

A.

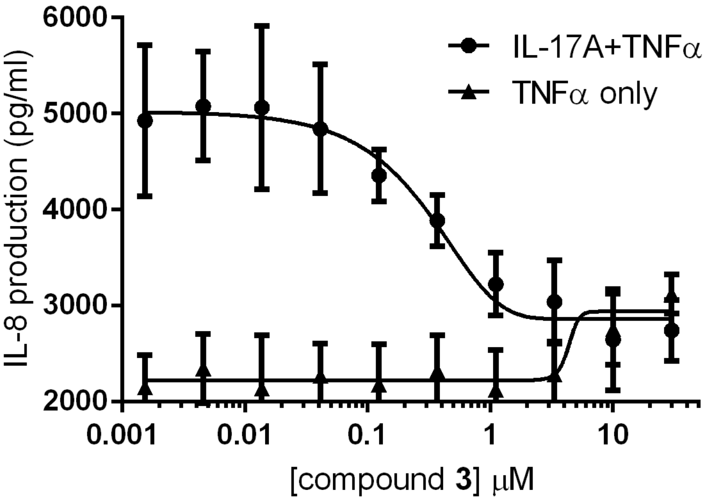

B.

Supplementary Figure S11. Compound **2** and **3** inhibit IL-8 production of keratinocytes synergistically stimulated by IL-17A and TNF-α, but not the baseline production stimulated by TNF-α alone. In this assay, 5 ng/ml IL-17A and 10 ng/ml TNF-α or 10 ng/ml TNF-α alone were added to the keratinocytes for IL-8 production. Data are mean and error bars of +/- standard deviation from 4 measurements. Notice the synergistic effects of IL-17A and TNF-α on IL-8 production, and compound **2** and **3** inhibition leads to TNF-α baseline level.
